# Microgel PAINT – nanoscopic polarity imaging of adaptive microgels without covalent labelling†
†Dedicated to Prof. Dr Ulrich E. Steiner (retired professor, University of Konstanz) for his 75th birthday.
‡
‡Electronic supplementary information (ESI) available. See DOI: 10.1039/c9sc03373d


**DOI:** 10.1039/c9sc03373d

**Published:** 2019-09-20

**Authors:** Ashvini Purohit, Silvia P. Centeno, Sarah K. Wypysek, Walter Richtering, Dominik Wöll

**Affiliations:** a Institute of Physical Chemistry , RWTH Aachen University , Landoltweg 2 , 52074 Aachen , Germany . Email: woell@pc.rwth-aachen.de

## Abstract

Polymer nanostructures have enormous potential for various applications in materials and life sciences. In order to exploit and understand their full capabilities, a detailed analysis of their structures and the environmental conditions in them is essential on the nanoscopic scale. With a super-resolution fluorescence microscopy technique known as PAINT (Points Accumulation for Imaging in Nanoscale Topography), we imaged colloidal hydrogel networks, so-called microgels, having a hydrodynamic radius smaller than the diffraction limit, gaining unprecedented insight into their full 3D structure which is not accessible in this much detail with any other experimental method. In addition to imaging of the microgel structure, the use of Nile Red as the solvatochromic fluorophore allowed us to resolve the polarity conditions within the investigated microgels, thus providing nanoscopic information on the *x*,*y*,*z*-position of labels including their polarity without the need of covalent labelling. With this imaging approach, we give a detailed insight into adapting structural and polarity properties of temperature-responsive microgels when changing the temperature beyond the volume phase transition.

## Abstract 2

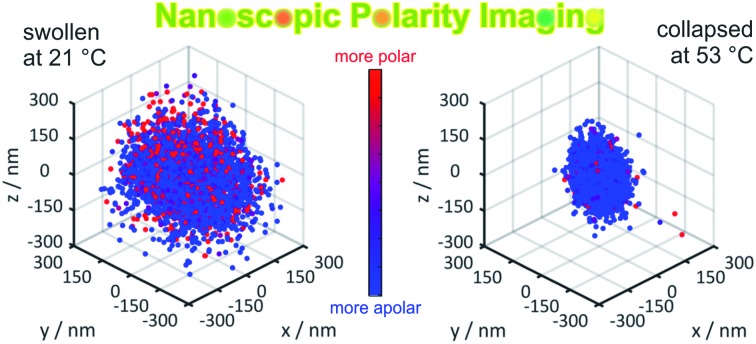
The 3D structure and the local environment of stimuli-responsive microgels were investigated with the superresolution fluorescence microscopy method PAINT using Nile Red as solvatochromic dye.

## Introduction

Microgels have received enormous attention in the last two decades and currently count among the most studied colloidal and polymer systems.^1^,^2^ Especially microgels that can change their shape or properties in response to external stimuli such as changes in temperature^3^,^4^ and pH,^5^,^6^ electrochemical triggers^7^,^8^ or light^9^ and, for multi-responsive microgels,^10^,^11^ combinations thereof^12^ bear huge potential for applications.^10^,^13^–^15^ For a deeper understanding of this switching behaviour and its consequences for applications, a thorough *in situ* investigation of concomitant changes in size, shape and polarity is essential. Cryogenic transmission electron microscopy (cryoTEM),^16^*in situ* TEM^17^ and atomic force microscopy (AFM)^18^ have proven to be suitable imaging techniques for soft matter. The low contrast without heavy atom staining, however, prevents exploitation of the full resolving power of electron microscopy, and AFM, despite its ability to probe rheological properties, is limited to information obtained by probing the microgel mechanically. The development of super-resolved fluorescence microscopy methods^19^–^22^ made also fluorescence microscopy suitable for visualizing microgels with diameters in the size range of hundreds of nanometers.^23^

Super-resolved fluorescence imaging of microgels has been restricted so far to a method called direct stochastic optical reconstruction microscopy (dSTORM)^24^ utilizing the chemical on- and off-switching of fluorophores induced by redox reactions with additives. With this technique, size changes of microgels due to co-nonsolvency were visualized^25^ and the selective labelling of different functionalizations in the core and shell, respectively, could be investigated.^17^ Furthermore, Conley *et al.* recently reported the compression and deformation of single microgels with increasing microgel concentration.^26^ Additives such as the switching buffer can be avoided when the structures are covalently labelled with photoswitches.^27^ However, in many cases covalent labelling requires significant efforts. Strategies to circumvent covalent labelling are of significant practical advantage since it allows super-resolution imaging to access a plenitude of polymer systems. Bergmann *et al.* found that non-covalent labelling of microgels with rhodamine 6G and subsequent imaging under typical dSTORM buffer conditions resulted in superresolved microgel images of high quality.^28^ An alternative to dSTORM, also without the need of covalent labelling, is superresolved Points Accumulation for Imaging in Nanoscale Topography (PAINT).^29^,^30^ Additionally, in contrast to dSTORM, the PAINT approach has the advantage that no switching buffer has to be added which might disturb the (polymer) system under investigation significantly. It has, for example, been used to study the structure of Large Unilamellar Vesicles (LUVs) where fluorescent probes continuously target the object of interest in a flux^29^ and been combined with other super-resolution methods.^31^ Additionally the technique is used for super-resolved imaging of DNA (DNA-PAINT).^32^ Furthermore, as an additional readout parameter, spectral information can be accessed.^33^–^35^ Bongiovanni *et al.*, for example, investigated the hydrophobicity of biological structures exploiting the solvatochromic behaviour^36^ of Nile Red in addition to the super-resolution imaging *via* PAINT.^37^ Local changes in the membrane polarity in live mammalian cells were investigated by employing Nile Red based SR-STORM and SR-PAINT by Moon *et al.*^38^ In general, super-resolution transient binding (STB) methods are emerging imaging techniques with high resolution, which avoid the limitation of photobleaching.^39^

Herein, we report on the PAINT approach to study the 3D structure and the point-wise polarity of thermo-sensitive core–shell microgel systems with super-resolution. In the model system investigated here, the core consists of poly(*N*-isopropylacrylamide) (PNIPAM) and the shell of poly(*N*-isopropylmethacrylamide) (PNIPMAM) with volume phase transition temperatures (VPTTs) of 32 °C and 42 °C, respectively. The polarity information is obtained by the use of the solvatochromic dye Nile Red,^40^ thus demonstrating an approach to gain polarity information along with the 3D structure.

## Experimental details

### Sample preparation

The synthesis of these core–shell microgels with the PNIPAM core and PNIPMAM shell has been previously published.^17^ 10 μL of 0.5 mg mL^–1^ of core–shell microgel solution was spin-coated onto a freshly plasma cleaned coverslip and placed inside a temperature cell (see the ESI‡ for more details). 100 μL of bidistilled water was added to the sample *in vitro*. In order to label the microgels non-covalently *via* the PAINT method, 2 μL of 10^–11^ M Nile Red solution in methanol was added.

### Experimental setup

For 3D PAINT measurements, the sample was excited by focusing a 488 nm laser beam onto the back focal plane of a 100× 1.3NA oil immersion objective lens (UPLFLN 100XO2, Olympus) in an inverted microscope (IX83, Olympus) using a plano convex lens of focal length 500 mm to obtain Köhler illumination. A single line dichroic mirror (zt 488 RDC, AHF analysentechnik, Tübingen) reflects the excitation beam and allows the emission from the sample to subsequently pass through an emission filter 510LP (AHF analysentechnik, Tübingen). The detection path consists of an imaging system made up of two plano convex lenses of focal lengths 100 and 200 mm for a two-fold magnification. In this study, we also make use of the solvatochromic behaviour of Nile Red in order to resolve the polarity of microgels at the nanoscale. In contrast to the studies by Ke Xu and coworkers,^35^,^38^ we did not refract the emission light through a prism, because we wanted to avoid additional spreading of the point spread functions that were already distorted by astigmatic imaging (see below). Instead, the emission was split into two channels using an Optosplit 2 bypass (CAIRN Research, UK). A dichroic filter (zt 594RDC, AHF analysentechnik, Tübingen) in the Optosplit reflects shorter wavelengths (<594 nm) and transmits longer wavelengths (>594 nm). Additionally, a 617/73 Brightline HC Bandpass emission filter (AHF analysentechnik, Tübingen) and a 514LP Razor edge emission filter (AHF analysentechnik) were introduced in the transmitted and reflected path of the Optosplit, respectively, to reduce the background. The different *z*-offsets in both the reflected and the transmitted channels were minimized with a corrector lens in each channel. For 3D imaging, a cylindrical lens of focal length 500 mm was introduced approximately 5 cm in front of the EMCCD chip of an Andor Ixon Ultra 897 camera. For all the PAINT measurements, a laser power density of 5.3 kW cm^–2^, an exposure time of 5 ms and electron multiplying mode setting with a gain of 200 were used. The number of frames recorded per area of the sample varied between 60 000 and 120 000. The images were recorded using a home-made temperature cell at six different temperatures: 21, 33, 35, 38, 43 and 53 °C. The temperature range was selected such that the VPTT of both, the PNIPAM core and PNIPMAM shell, could be covered.

The recorded movies were separately cropped for the shorter and the longer wavelength channels of the Optosplit and analysed with ThunderSTORM.^41^ The cropped movies and the super-resolved localization files were subsequently fed as inputs to custom-made MATLAB Optosplit routines (see the ESI‡ for more details). The output obtained from the Optosplit routines contains the *x*, *y*, *z* positions of labels and the ratio of the emission intensity between the longer and the shorter wavelength channels. Details on this intensity ratio and its calibration to compare it to the conditions in different solvents can be found in the ESI.‡ The localization files are subsequently fed into VISP software^42^ where the localizations of individual microgels are cropped and the 3D distribution of localizations is plotted using a custom-made MATLAB routine (see the ESI‡).

## Results and discussion

**Fig. 1:**
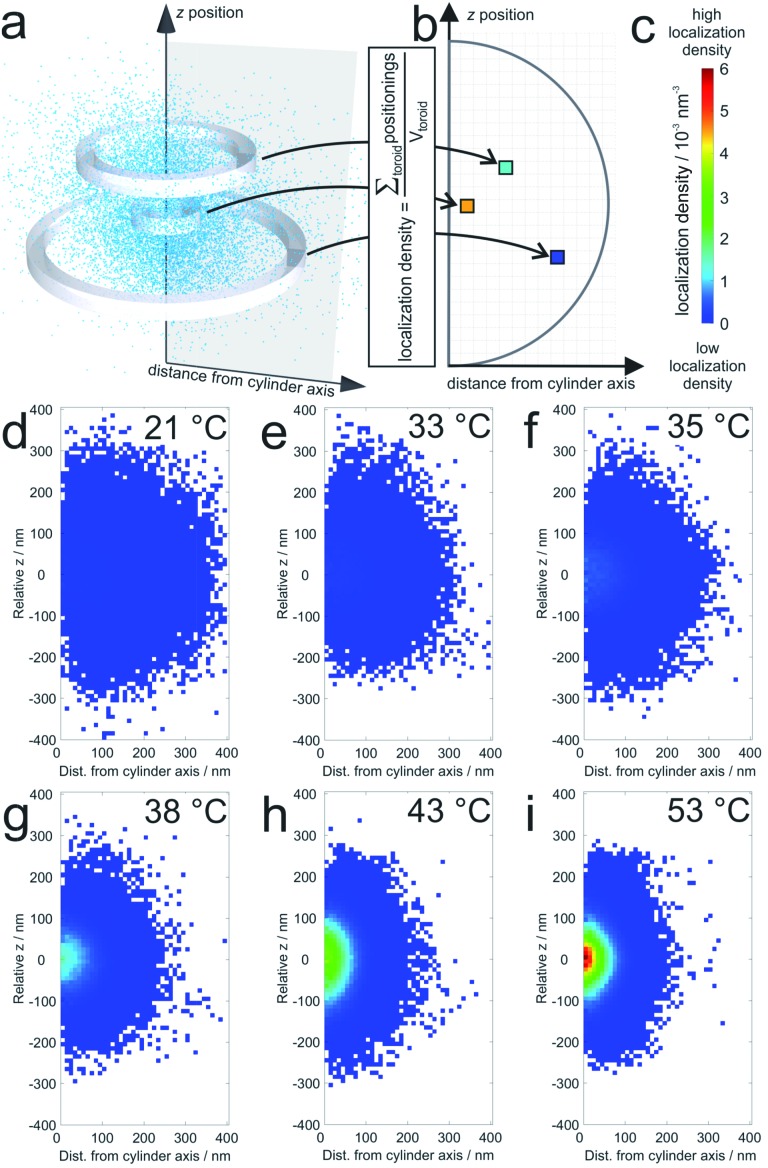
Average localization densities of 20 microgels: (a) example for the procedure for calculation of the labelling densities at different distances from the cylinder symmetry axis (for a single collapsed microgel at 53 °C). The sum of localizations within one toroid is divided by the volume of the respective toroid in the graph in (b); (c) shows the colour scale for the localization densities in localizations per nm^3^ which are shown for (d) 21 °C, (e) 33 °C, (f) 35 °C, (g) 38 °C, (h) 43 °C, and (i) 53 °C. White pixels mean that no localizations were detected in the corresponding toroid. For pixels close to the symmetry axis, this is sometimes observed in particular in the pole regions. It should be noted that the sampling density of the localizations at the periphery is significantly lower than that in the center (see also Fig. 4).

The average localization densities of 20 microgels at six different temperatures are shown in Fig. 1. For better clarity, we present the axially symmetrical data in a graph where the averaged densities are plotted *versus* a relative *z*-position and the distance from the symmetry axis (see Fig. 1a). At room temperature, the density of localizations is rather constant throughout the microgel and the sparse pixels from the outer regions represent localizations close to dangling chains of the swollen microgels. Additionally, some localizations in this region can be due to localization inaccuracies. At 33 °C and 35 °C, respectively, it can be observed that the PNIPAM core starts to collapse since the density of localizations in the centre starts to increase gradually with the increase in temperature. Also the overall size of the microgels decreases with respect to room temperature. At higher temperatures, which reach beyond the VPTT of the shell, the entire microgel becomes more compact. Still, the labelling density in the core remains significantly higher than that in the shell.

**Fig. 2:**
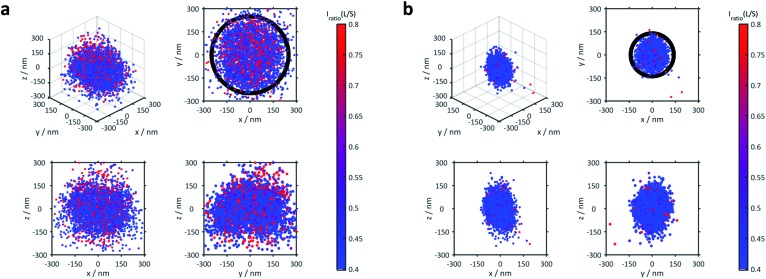
3D distribution of localizations of a single representative microgel (a) at 21 °C and (b) at 53 °C, respectively. A full 3D view and projections to the *xy*, *xz* and *yz* planes are shown. The colour code represents the solvatochromism of each single localized fluorophore expressed using the ratio between the long wavelength (>594 nm) and the short wavelength (<594 nm) channel with scaling from 0.4 to 0.8 (see the colour bar). The circle in the *xy*-projection represents the hydrodynamic radius of the microgel determined *via* DLS which is 250 nm for (a) and 140 nm for (b).

The 3D distribution of Nile Red labels of a single microgel along with *xy*, *yz* and *xz* projections for the lowest (21 °C) and the highest temperatures (53 °C), respectively, is shown in Fig. 2. The sizes of the microgels obtained *via* PAINT are in agreement with those of the DLS measurements at the respective temperature denoted by the black circle in the *xy* projection (see the ESI of Gelissen *et al.*^17^). In the *z*-direction, a slight elongation can be observed which originates from the lower localization accuracy of approx. 60 nm in the *z*-direction in contrast to approx. 20 nm in the *x*- and *y*-directions. Since the coverslip surface cannot be detected with our method, we set the *z*-axis to zero at the centre of the microgel. Conclusively, the position of the coverslip is always at negative *z*-values. In addition to the localizations, the polarity of each position is represented using its colour. The colour of the points indicates the intensity ratio between the long wavelength (>594 nm) and the short wavelength (<594 nm) channel which, in Fig. 2, is scaled from 0.4 to 0.8. Throughout our paper, this intensity ratio representing the polarity of the corresponding positions will be called their solvatochromic value. As shown in Fig. 2 and Fig. S2 of the ESI,‡ the environment in the microgels is in general more polar in the swollen state below the VPTT. In the collapsed state, the fluorescence emission of almost all Nile Red labels is hypsochromically shifted as expressed using the lower solvatochromic value.

**Fig. 3:**
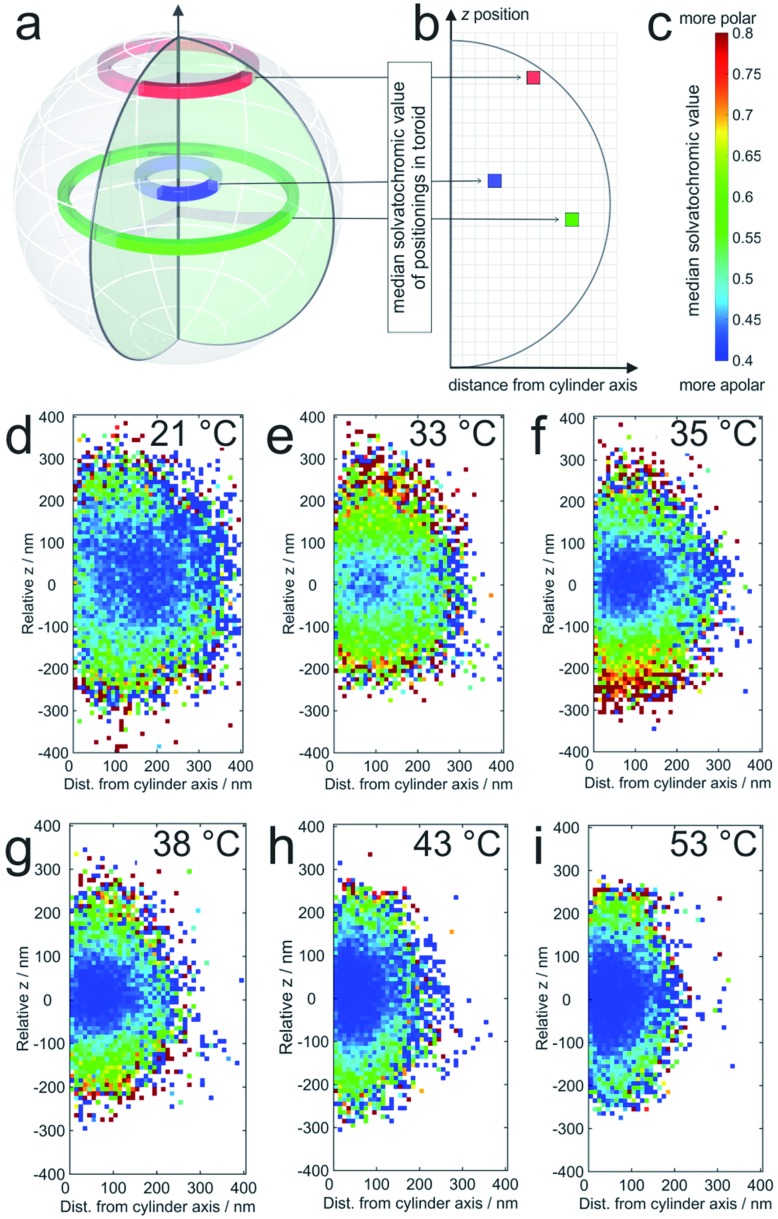
(a) The solvatochromic values are calculated from the medians of the corresponding values of all localizations within one toroid as presented in three examples; (b) the median solvatochromic values are plotted in pixels representing the distance from the cylinder axis and coloured with values shown in (c). The colours represent the solvatochromic values expressed by the ratio between the long wavelength (>594 nm) and the short wavelength (<594 nm) channel with scaling from 0.4 to 0.8. Solvatochromic values averaged over 20 microgels at (d) 21 °C, (e) 33 °C, (f) 35 °C, (g) 38 °C, (h) 43 °C, and (i) 53 °C. White pixels mean that no localizations were detected in the corresponding toroid. For pixels close to the symmetry axis, this is sometimes observed in particular in the pole regions. It should be noted that the sampling density of the localizations at the periphery is significantly lower than that in the center (see also Fig. 4).

The averaged super-resolved solvatochromic image at different temperatures is shown in Fig. 3. Compared to Fig. 1, in which the localization density is shown, Fig. 3 connects each pixel to its solvatochromic value ranging from 0.4 to 0.8. The pixel-wise values are obtained from the median values of the respective values of all localizations within the corresponding concentric ring. At 21 °C (Fig. 3d) the core and shell are completely swollen. The solvatochromic values corresponding to the inner part of the microgel account to values ranging from 0.4 to 0.6 (blue to green). In the outer part of the microgel, values of around 0.8 are observed (red), due to the more polar surroundings or even to the so-called dangling polymer chains.

At 33 °C (Fig. 3e), a temperature just slightly above the VPTT of the core, the size of the point cloud decreases and the solvatochromic values close to the centre are shifted towards lower values (approximately between 0.4 and 0.55), indicating that the core is more apolar after the collapse. Interestingly, the collapse of the core induces increased solvatochromic values in the shell. It seems that the core collapse induced structural changes in the surroundings of Nile Red labels adsorbed to the shell, a fact that is also highly important for microgels as encapsulating agents, for example, for drug delivery and for their application in catalysis.^43^,^44^ Especially close to the coverslip, we observe a significantly higher polarity. This increased polarity is reasonable since the coverslips are slightly negatively charged due to our surface cleaning procedure (see the ESI‡). The contrast between the core and shell is consistently observed for the measured temperatures between the VPTT of the core and that of the shell. Also, the size of the point clouds, and thus of the microgels, decreases gradually with increasing temperature (see Fig. 3d–i). Increasing the temperature above the VPTT of the shell at *ca.* 42 °C also causes collapse of the PNIPMAM polymer chain shell. Consequently, at this point the shell becomes rather hydrophobic and the solvatochromic values within the shell decrease to values below 0.55, similar to the values in the core. Only some of the solvatochromic values in the most outer regions of the microgels remain rather high. At 53 °C, the highest measured temperature, we observe the smallest size and the highest overall hydrophobicity of the microgels.

In general, it should be noted that the Nile Red molecules observed in the microgels presented here are in surprisingly apolar surroundings as is obvious from their solvatochromic values. The local environment of the majority of Nile Red labels can be compared to that ranging between *n*-decane and ethylacetate (for calibration see the ESI‡). The reason for this could be that the Nile Red molecules cause local changes of the polymer conformation around them. These changes, however, do not affect the microgel properties due to the sub-nanomolar concentration of the dye.

**Fig. 4:**
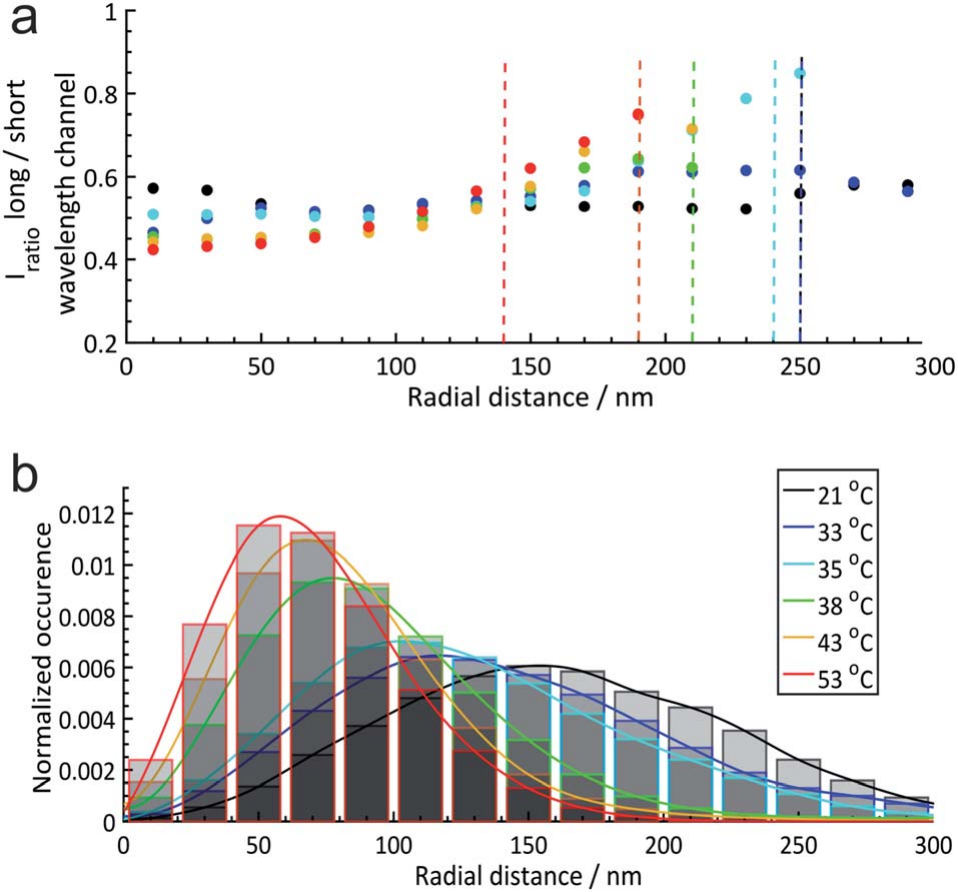
(a) Radial solvatochromic values of core–shell (PNIPAM core and PNIPMAM shell) microgels at 6 different temperatures: 21 °C, 33 °C, 35 °C, 38 °C, 43 °C, and 53 °C. Dashed lines indicate the corresponding hydrodynamic radii determined *via* DLS measurements. In this plot, the hydrodynamic radii estimated by dynamic light scattering (DLS) at each temperature have been included as a vertical dashed line (the blue and black dashed lines overlap at 250 nm). (b) Normalized radial distributions of the microgels at different temperatures. The coloured lines are a guide for the eye. More details can be found in Section 6 of the ESI.‡

For further discussion, we reduced the full 3D information to radial distributions assuming the spherical symmetry of the microgels which is a reasonable assumption as shown in the images in the figures above. Based on this simplification, in Fig. 4 we plotted the median solvatochromic values and the localization densities *versus* the radial distance from the centre of microgels at different temperatures for 20 to 30 microgels.

Fig. 4 emphasizes the trends discussed above that the median solvatochromic values are rather constant at 21 °C when the polymers, both in the core and in the shell, are in the swollen state. Collapse of the core results in lower polarity in this region, which surprisingly causes a shift to higher solvatochromic values and thus an apparent higher polarity in the shell. We attribute this to a change in polymer conformation in the shell.^45^ At 53 °C, where the core and shell are collapsed, the lowest solvatochromic values are observed throughout the microgel. Only in the most outer parts of the shell, the values increase even beyond the ones at lower temperatures. Presumably, the Nile Red labels are in very low polymer density regions mainly surrounded by water.

**Fig. 5:**
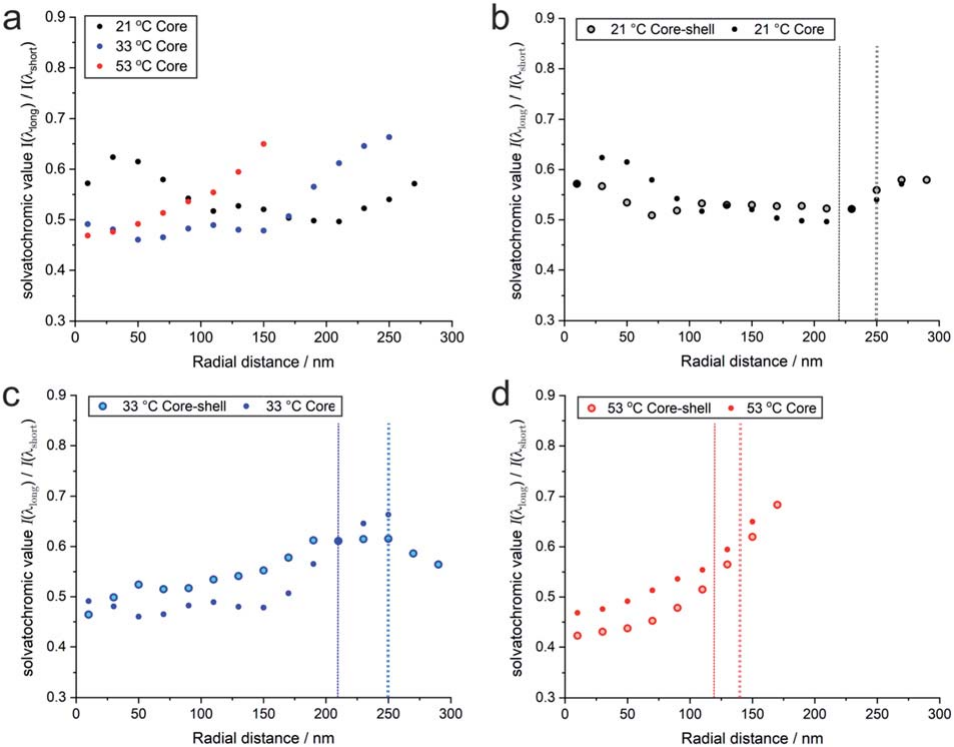
(a) Radial solvatochromic values for PNIPAM-core-only microgels at three selected temperatures, and a comparison of the values with the corresponding values for PNIPAM-core–PNIPMAM-shell microgels at (b) 21 °C, (c) 33 °C, and (d) 53 °C. The data points represent median values of rather broad distributions. Their detailed statistics is presented as boxplots in Fig. S20 of the ESI.‡

It is also interesting to compare these results with the solvatochromic values of a microgel which consists only of the PNIPAM core before polymerizing the shell onto it (for detailed analyses see also the ESI‡). As shown in Fig. 5, the solvatochromic values inside the microgels at *r* < 100 nm are significantly lower in the core–shell microgel and equalize at larger distances from the centre. After increasing the temperature to 33 °C, all solvatochromic values decrease. However, at this temperature, the polarity inside the microgel is lower throughout the core-only microgel. At 53 °C, where all polymers are collapsed, the solvatochromic values of the core–shell microgel are significantly lower. At this temperature, both the core–shell and the core-only microgels show a similar increase in polarity in vicinity of their hydrodynamic radii. The comparison of the temperature-dependent behaviour of the core–shell and core-only microgels shows that the shell has a significant influence on the core when considering polarity. At 21 °C, the shell makes the core more apolar than the core alone. Since the shell was synthesized onto the core in its collapsed state at elevated temperature, the conformation at room temperature presents additional stress, especially in the core which is more apolar than the same core without the shell. When the core collapses at *ca.* 32 °C, it cannot reach the fully collapsed state due to the conformational restrictions opposed by the shell. At 53 °C, when both the core and shell are collapsed, the polarity in the core–shell microgel is below the values of the core-only microgel. In both cases, however, the polarity increases at the periphery of the microgels.

**Fig. 6:**
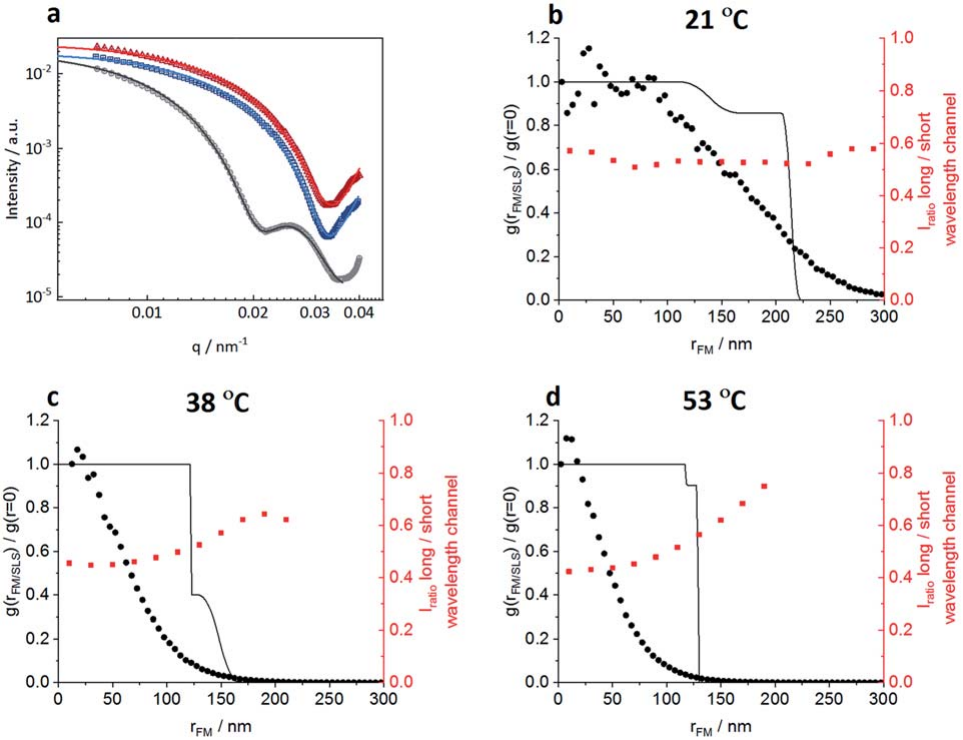
Comparisons of radial distribution of polymer density within the microgels from static light scattering (SLS) with the radial distribution of Nile Red localizations from 3D PAINT measurements of surface-immobilized microgels. (a) SLS scattered intensities of the microgels fitted with a fuzzy-core-shell-model at 3 different temperatures: 21 °C (black curve), 38 °C (blue curve), and 53 °C (red curve). (b–d) The radial density of polymers within the microgel obtained by the fit of the SLS intensities (black line), the distribution of Nile Red localizations from 3D PAINT averaged over 20 microgels (black filled circles) and the radial evolution of the solvatochromic values is presented for (b) 21 °C, (c) 38 °C, and (d) 53 °C.

In order to correlate the solvatochromic values with polymer densities, we compare the localizations and their solvatochromic values from PAINT with the radial distribution of polymers within the microgels obtained by fitting the static light scattering (SLS) intensities at different temperatures, Fig. 6. It is worth noting that while SLS probes the polymer density within the microgels, PAINT probes the distribution of localizations of the dye diffusing within the microgels. These two quantities are not the same but offer complementary information on the structure of the microgels. The SLS intensities in Fig. 6a were fitted with a fuzzy-core–shell-model (see the ESI‡ for more details on the fitting parameters).^46^ This is the simplest model that allows obtention of good fits and values of the microgel radius consistent with the hydrodynamic radius obtained by DLS. Due to the high quality of the fits and the consistency of the obtained radial distribution of polymers shown with black lines in Fig. 6b–d with DLS data, more complex models with a higher number of parameters are not needed.^28^,^47^ The radial distribution of polymers within the microgel is compared to (i) the respective radial distributions of localizations obtained by PAINT measurements averaged over 20 microgels (black dotted line) as also outlined in the study by Siemes *et al.*^48^ and (ii) to the median solvatochromic values (red dotted line).

At 21 °C, the localization density distribution is similar to the polymer distribution within the core but gradually decreases towards the outer microgel regions whereas the polymer density in the shell shows only a small drop. The median solvatochromic values remain rather constant. It seems that neither the polymer density nor the polarity as probed by Nile Red changes significantly. Still Nile Red fluorescence appears more often in the inner microgel region. The reason for this is probably the higher cross-linker density in the core which increases the probability of Nile Red to become immobile.

As shown in Fig. 6c, at 38 °C, SLS data show that the core is collapsed and hence has higher polymer density when compared to the still swollen shell in the radial distance range of 125 nm to 170 nm. The radial distribution of localizations is the highest close to the centre and then decreases rapidly. Only a few localizations appear in the low-density shell region. These localizations have significantly higher solvatochromic values. As already discussed above with respect to Fig. 3, the collapse of the core seems to change the network structure in the shell in a way that it becomes more polar, thus reducing the probability of Nile Red adsorption and increasing the local solvatochromic values.

Fig. 6d presents the data at 53 °C when the core and shell are collapsed. Surprisingly, even here, the localizations drop rapidly from the core towards the periphery and the solvatochromic values in the now collapsed shell remain increased. Our findings point to a situation in which the shell collapses, as indicated by SLS, but despite the collapse, heterogeneities in the polymer network structure exist which result in higher probability of Nile Red molecule adsorption in the centre. Additionally, the polarity inside the collapsed shell is higher than that in the core. Presumably, the shell cannot find its optimal collapse conformation due to the restrictions of the previously collapsed core. It is worth noting that combining the complementary information obtained from SLS (polymer radial density profile) with PAINT (radial density of localizations) provides more insight into the structural changes within the microgels. The fact that scattering and PAINT probe different quantities allows for a more complete characterization of the microgels.

All of our findings also show that small molecules can diffuse through hydrogel networks in the swollen and even in the collapsed state. This is demonstrated with Nile Red which is localized in the microgel core even at 53 °C where the core and shell are both collapsed. This result has to be taken into account, for example, for the design of microgels as drug delivery systems and for catalysis.^43^,^44^

## Conclusions

We analysed the nanoscopic 3D structure and local polarity conditions in thermo-responsive core–shell microgels with a localization-based super-resolution fluorescence microscopy approach which does not require covalent labelling. The polarity was accessed using the solvatochromic dye Nile Red. The temperature-dependent change of the structure of the microgels and the polarity within them were obtained. The size changes of the microgels are in good agreement with static and dynamic light scattering data. The polarity decreases with the collapse of the respective microgel compartment. We are convinced that, beyond its power to resolve nanoscopic polymer structures, the presented PAINT approach can give unprecedented insights into the environmental conditions within such polymer structures on the nanoscale. In our study, we found that the majority of Nile Red labels reported surprisingly apolar local surroundings. Such information can currently not be obtained by any other experimental technique. Not only does it present a novel method to analyse compartments of synthesized nanostructures, but it is essential, for example, for the design of drug delivery systems, for understanding the performance of microgels in extraction and separation processes and for their application in multi-step catalysis.

## Conflicts of interest

There are no conflicts to declare.

## Supplementary Material

Supplementary information

Supplementary movie

Supplementary movie

Supplementary movie

Supplementary movie

Supplementary movie

Supplementary movie

Supplementary movie

Supplementary movie

Supplementary movie

Supplementary movie

Supplementary movie

Supplementary movie

Supplementary movie

Supplementary movie
