# Supplementary material for: Microgel PAINT – nanoscopic polarity imaging of adaptive microgels without covalent labelling
Source: Chem Sci. 2019 Sep 20;10(44):10336–42. doi: 10.1039/c9sc03373d (PMC6984396; doi:10.1039/c9sc03373d)
Supplement: Supplementary file 1 [file SC-010-C9SC03373D-s001.pdf]

Electronic Supplementary Material (ESI) for Chemical Science.
This journal is © The Royal Society of Chemistry 2019

**Supplementary Information:**

# Microgel PAINT — Nanoscopic Polarity Imaging of Adaptive Microgels without Covalent Labelling

Ashvini Purohit,[1] Silvia P. Centeno*,[1] Sarah K. Wypysek,[1] Walter Richtering,[1] and Dominik Wöll*[1]
[1] *Institute of Physical Chemistry, RWTH Aachen University, Landoltweg 2, 52074 Aachen, Germany*

## 0. Contents

1. Calibration measurements 2
   1.1. Positioning and point registration between wavelength channels 2
   1.2. $z$ Calibration measurements 2
2. Analysis of the data 3
   2.1. Processing the raw data using ThunderSTORM 3
   2.2. Optosplit routines in MATLAB 3
   2.3. Visualization and plotting of single microgels 3
3. Temperature cell and the objective heating 3
4. Protocol for cleaning glass substrates 4
5. Microgels used for PAINT measurements 4
6. 3D localization distributions at different temperatures of core-shell microgels 4
7. Calibration of intensity ratio versus emission maxima of Nile Red in different solvents 8
8. Temperature dependent fluorescence spectra of Nile Red in different solvents 9
9. Non-normalized fluorescence spectra of Nile Red in different solvents at different temperatures 12
10. SLS measurements 14
11. PAINT measurements on core-only microgels 16
12. Comparison of the core-shell-microgels with the corresponding core-only microgels 19
    12.1. Boxplot 20
13. Supporting movies 21

References 21

# 1. Calibration measurements

## 1.1. Positioning and point registration between wavelength channels

The emission of Nile Red was split into 2 wavelength channels ($\lambda\ > 594$ nm and $\lambda\ < 594$ nm) by employing an Optosplit II LS (AHF analysentechnik). The corresponding positions in both the wavelength channels had to be registered to each other. For this purpose 20 µl of a 1 mM water dispersion of TetraSpeck™ Microspheres (0.1 $\mu$m, blue/green/orange/dark red fluorescence, ThermoFisher scientific) was spincoated onto a freshly plasma-cleaned coverslip. To cover the entire field of view, a movie of 1071 frames was recorded while the sample was scanned in a snake like pattern in $x$ and $y$ directions with 1 µm steps. A registration matrix was calculated which correlates the coordinates of channel 1 to the corresponding coordinates of channel 2.

## 1.2. $z$ Calibration measurements

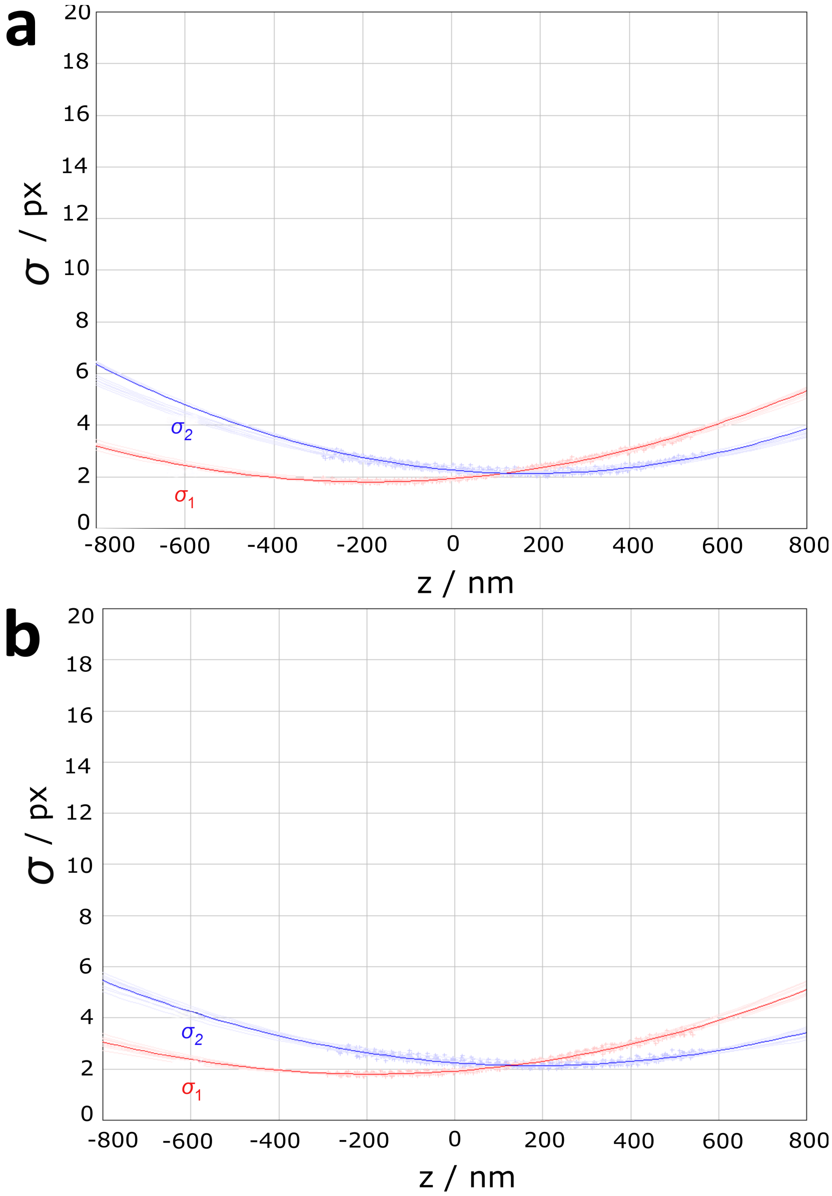


**Figure S1:** a) z calibration curve in channel 1 – shorter wavelength channel (< 594 nm) and b) z calibration curve in channel 2 – longer wavelength channel (> 594 nm) obtained from ThunderSTORM.

For 3D astigmatic imaging, a $z$ calibration measurement was performed to determine the dependency of the widths $\sigma_x$ and $\sigma_y$ of a Gaussian fit to the astigmatic point spread function on the $z$ position of the molecule.[1] For the measurements, 20 µl of a $2 \times 10^{-4}$ M water dispersion of TetraSpeck™ microspheres (Diameter = 0.1 µm, blue/green/orange/dark red fluorescence, ThermoFisher scientific) dissolved in bi-distilled water was spincoated onto a freshly plasma-cleaned coverslip. The beads are scanned in $z$ direction by moving the piezo stage with a step size of 20 nm for a $z$ range of 1 µm. The $z$ calibration files were analyzed separately for both the channels in order to correct for the $z$ offset. The 3D calibration data were analysed with a ImageJ plugin called ThunderSTORM. The calibration curves are shown in Figure S1.

# 2. Analysis of the data

## 2.1. Processing the raw data using ThunderSTORM

The 3D super-resolution results were obtained by analysis with ThunderSTORM, an ImageJ plugin. The batch analysis crops the movies into the 2 channels of the Optosplit and then perform the 3D positioning for both channels with their respective $z$ calibration curves. This way, errors introduced by the $z$ offset in both the channels can be avoided. The results obtained consist of 3D positions and their respective intensities in both channels separately.

## 2.2. Optosplit routines in MATLAB

The intensity ratio between the emission intensity in the longer and shorter wavelength channel was determined for each localization. The results from the ThunderSTORM analysis are fed into custom-made Matlab-routines. They are processed in four steps: (1.) $xy$-calibration of the 2 channels; (2.) pair-finding of the localized cropped movies, and determination of the intensity ratios of longer to shorter wavelength channels; (3.) sorting of the localized pairs according to localizations found in both the channels, localizations found only in the shorter wavelength channel and localizations that were only found in longer wavelength channel; (4.) microgel positions are determined and their centres of mass and radii are set. With these positions, the median values for about 30 to 40 microgels was determined and the median values were plotted with respect to radius and the intensity ratios. To visualize the distribution of localizations at different temperatures, radial distribution plots are presented.

## 2.3. Visualization and plotting of single microgels

The superresolved localization files with $x$, $y$, $z$ coordinates and intensity ratio columns of all the microgels are analyzed with custom made MATLAB routines wherein the density distributions and the 3D distributions of localizations with the corresponding and $xy$, $yz$, $xz$ projections of individual microgels are plotted.

# 3. Temperature cell and the objective heating

A custom made temperature cell with a peltier element on top is used for heating the sample between 21 °C to 53 °C. In order to avoid the temperature gradient between the sample and the objective lens of the microscope, an objective heater (Okolab: OBJ-COLLAR-2532 with H401-T-CONTROLLER) is used and maintained at the same temperature as that of the temperature cell.

# 4. Protocol for cleaning glass substrates

For fluorescence microscopy measurements it is important to have clean glass substrates to avoid unnecessary background levels arising because of the unclean coverslips. The glass substrates used for PAINT super-resolution imaging are circular glass coverslips of diameter 40 mm and thickness 0.19 mm (Menzel Gläser, Thermo Scientific, Germany). The coverslips were immersed in 1 % Hellmanex III (Hellma GmbH & Co.KG, Müllheim/Germany) solution overnight. Then the coverslips are washed thoroughly with approximately 5 L of Milli-Q water to remove any residual salt and kept overnight in an oven at 60 °C for drying . Before the measurements, the dried coverslips are air plasma cleaned for 15 minutes on both the sides of the coverslips employing a Femto plasma cleaner (Diener electronic GmbH + Co. KG). The glass coverslips are negatively charged after plasma cleaning which enables the microgels to adsorb well onto the coverslips.

# 5. Microgels used for PAINT measurements

Core-only PNIPAM microgels (PID: 21.11102/9ff9488a-95c0-443c-92cc-e8acf08dfad7, SFB 985 sample repository) were synthesized by Alex Oppermann.[2] For the synthesis, 92% NIPAM (N-Isopropylacrylamide),5% BIS (N,N'-Methylenbisacrylamide) as cross-linker, 3% APMH (N-aminopropylmethacrylamide hydrochloride) as co-monomer, and $<$ 1% AAPH(2,2'-azobis(2-methyl-propionamidine)·2 HCl) (V50) as the initiator were used.

Core-shell microgels with PNIPAM core (same as as in the core-only microgels) and PNIPMAM shell (PID: 21.11102/7abc5c6a-1203-4c1c-ae53-8c45229ea332, SFB 985 sample repository) were also synthesized by Alex Oppermann. Core of these microgels is made up of same components as in case of core only microgels. Shell is made up of 95.5% NIPMAM, 3% BIS, 1.5% DSDMA (bis(2-methacryloyl)oxyethyl disulfide) as co-monomer, and $<$ 1% AAPH(2,2'-azobis(2-methyl-propionamidine)·2 HCl)( v50) as the initiator.

The polydispersity index (PDI) of the microgels was determined by cumulant fitting of the dynamic light scattering data (DLS) and also from the static light scattering (SLS) data by considering the ratio of standard deviation and the mean radius obtained by the Fuzzy core-shell Model fit by importing the form factors to FitIt! (O. L. J. Virtanen, FitIt!, 2015. https://www.github.com/ovirtanen/fitit). Listed below in table S1 are the polydispersities of the two types of microgels at 21 °C.

**Table S1:** Polydispersity indices (PDI) of the microgels

| Microgel type | PDI from DLS in % | PDI from SLS in % |
|---|---|---|
| Core only | $7.6 \pm 4$ | - |
| Core-Shell | $12.6 \pm 4$ | $12.6 \pm 1$ |

More information on the synthesis of core only and core-shell microgels and their dynamic light scattering measurements can be found in the Supporting Information of previously published research.[2]

# 6. 3D localization distributions at different temperatures of core-shell microgels

Figures S2-S4 show the 3D distribution of localizations of individual core-shell microgels. The colour represents their point-wise solvatochromic value, i.e. the intensity ratio of longer and shorter wavelength channel. Along with the 3D distribution, $xy$, $yz$ and $xz$ projections of single microgels at different temperatures are also included. The sizes of the distributions obtained from PAINT measurements are in good agreement with the hydrodynamic radii obtained by dynamic light scattering. An increase of temperature results in a gradual decrease of the size of the microgel and of the solvatochromic values. This spectral blue shift with increasing temperature implies lower polarity at elevated temperature.

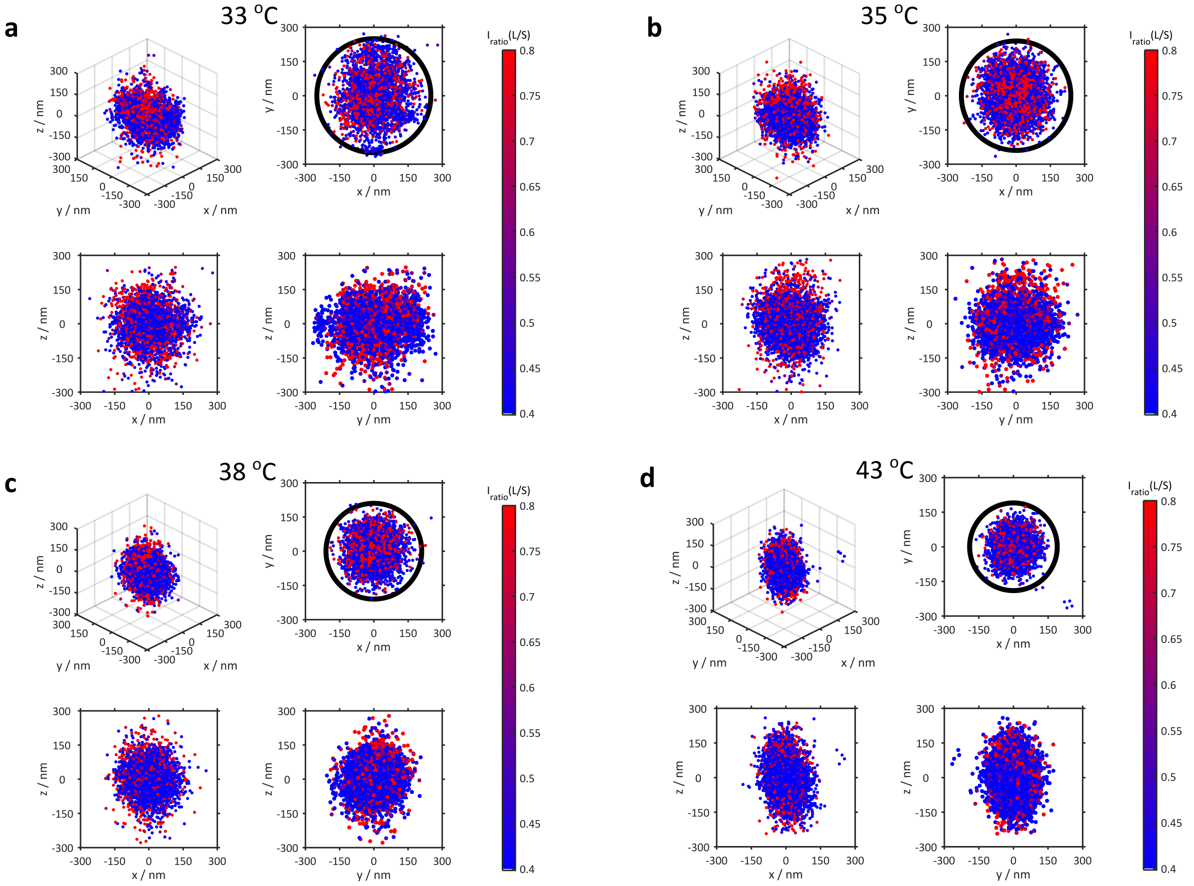


**Figure S2:** 3D distribution of localizations of individual core-shell microgels coloured according to their their point-wise solvatochromic value. The 3D distributions are presented in the upper left corners. Additionally, $xy$, $yz$ and $xz$ projections of single microgels are shown. The sizes of the distributions obtained from PAINT measurements are in good agreement with the hydrodynamic radii obtained by dynamic light scattering as presented by the circle in the $xy$ projection.

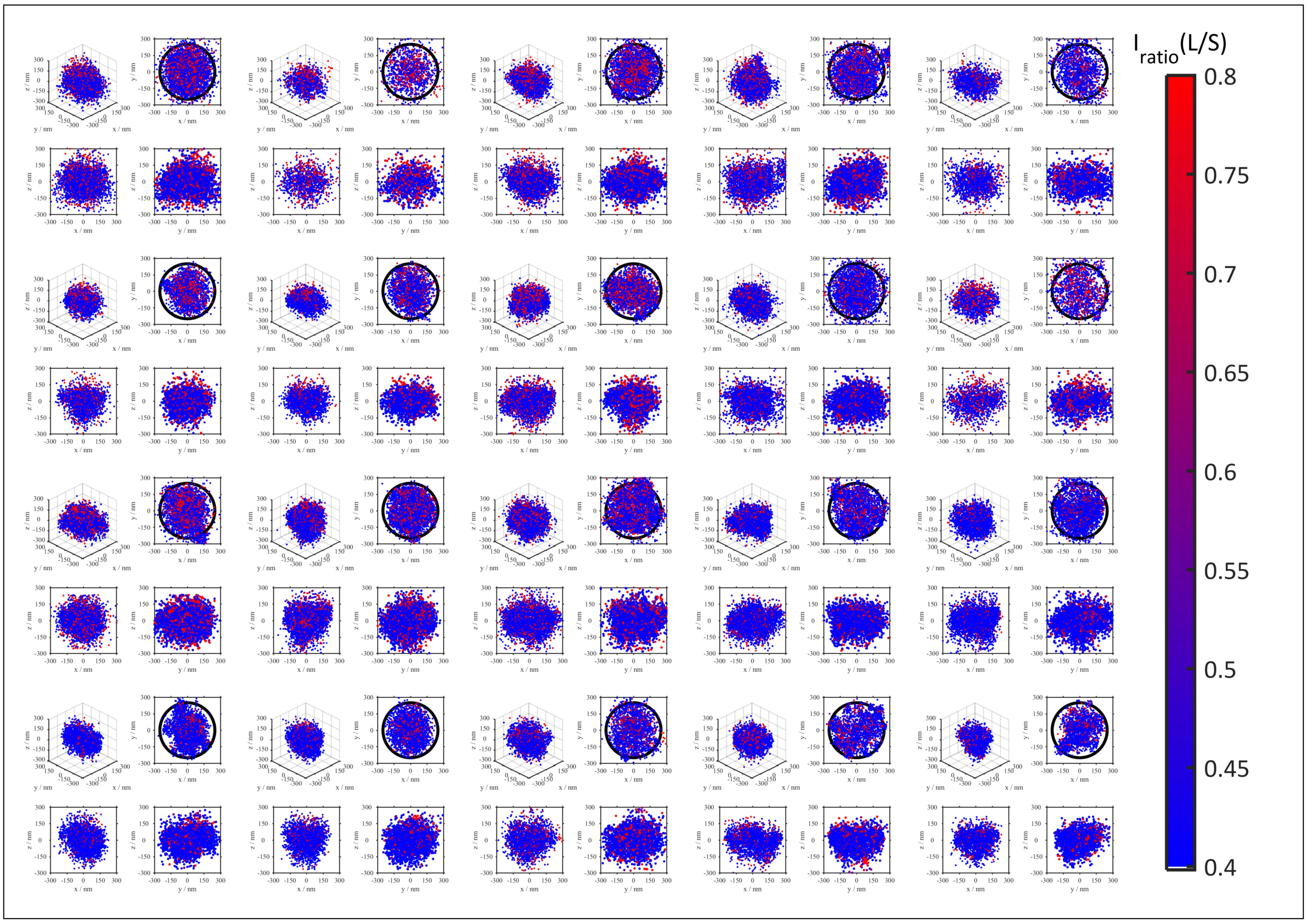


**Figure S3:** 3D distribution of localizations of 20 individual core-shell microgels at 21 °C coloured according to their their point-wise solvatochromic value. The 3D distributions are presented in the upper left corners. Additionally, *xy*, *yz* and *xz* projections of single microgels are shown. The sizes of the distributions obtained from PAINT measurements are in good agreement with the hydrodynamic radii obtained by dynamic light scattering as presented by the circle in the *xy* projection.

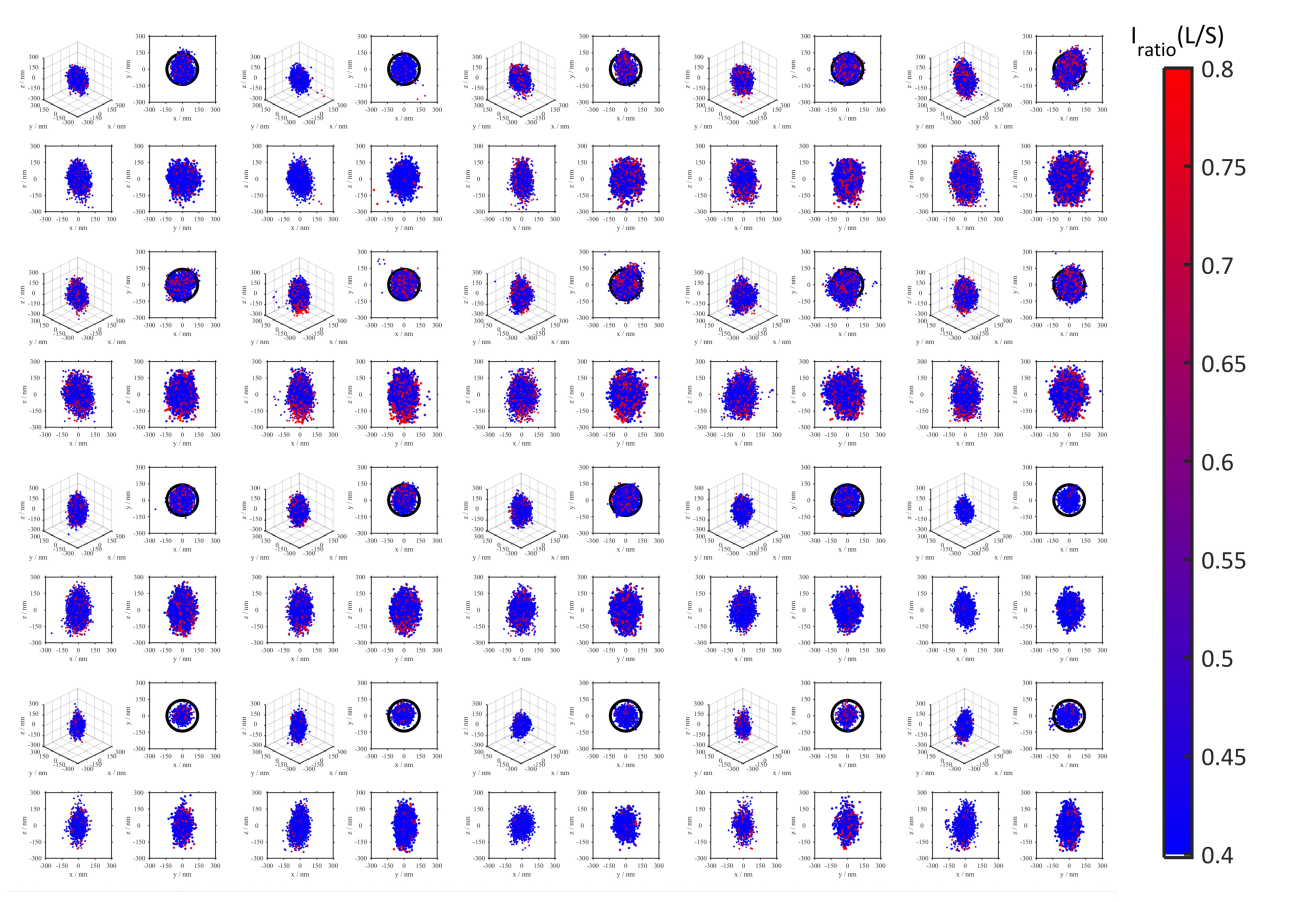


**Figure S4:** 3D distribution of localizations of 20 individual core-shell microgels at 53 °C coloured according to their their point-wise solvatochromic value. The 3D distributions are presented in the upper left corners. Additionally, $xy$, $yz$ and $xz$ projections of single microgels are shown. The sizes of the distributions obtained from PAINT measurements are in good agreement with the hydrodynamic radii obtained by dynamic light scattering as presented by the circle in the $xy$ projection.

## 7. Calibration of intensity ratio versus emission maxima of Nile Red in different solvents

In order to connect the intensity ratios measured when splitting the emission light using the Optosplit to chemical environments we calibrated them with the following two independent methods:

1. The first method was a direct calculation from the emission spectra of Nile Red (obtained from Sigma Aldrich) in different solvents (all spectroscopic grade). These spectra were obtained on a Jasco FP6500 fluorescence spectrometer with an excitation wavelength of 488 nm. They were corrected with the appropriate wavelength-dependent apparatus function of the spectrometer using the *Calibration Kit* Spectral Fluorescence Standards of the Federal Institute for Materials Research and Testing (BAM, Berlin, Germany) and the software LINKCORR. The fluorescence spectra (see Figure S6 top) were multiplied with the product of the transmissions of all filters (see Figure S6 bottom) placed in long and the short wavelength path of the microscope, respectively. The corresponding emissions of the long and short wavelength filter combination are highlighted by the filled curves. In Figure S5, the intensity ratio of the transmissions of the spectra with respect to the filters is plotted as red open circles versus the wavelength of fluorescence maxima obtained from the spectra.

2. The second calibration method was directly performed with the Optosplit and the widefield fluorescence microscopy system, which was also used for the PAINT measurements. A drop of Nile Red solutions in the corresponding solvent was placed onto a coverslip on the microscope. The focus was set to 10 $\mu$m in solution to avoid any possible interface effects. The emission signal in both channels were recorded on an ANDOR Ixon 897 EMCCD camera. The average intensities in the short wavelength channel was obtained by averaging of an 10 x 10 pixel area around the maximum intensity in the field of view. The same procedure was performed in the long wavelength channel with the corresponding region of interest known from the calibration procedure described in section 1. Both regions were corrected for background obtained by a measurement of pure solvent under otherwise identical conditions. The ratio of these corrected intensity ratio are plotted versus the wavelength at the intensity maximum as obtained by the fluorescence spectra in Figure S5 as solid blue circles.

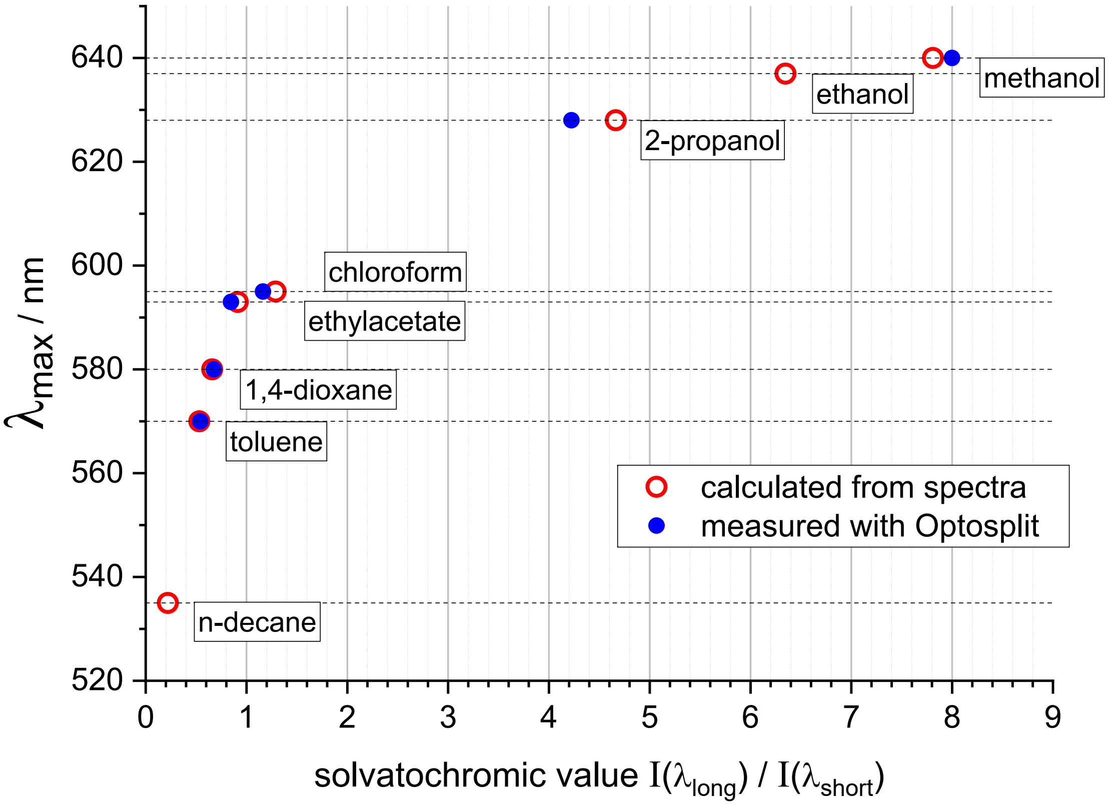


**Figure S5:** Calibration of the correlation of the intensity ratio between long and short wavelength channel for the two different calibration methods described in the text.

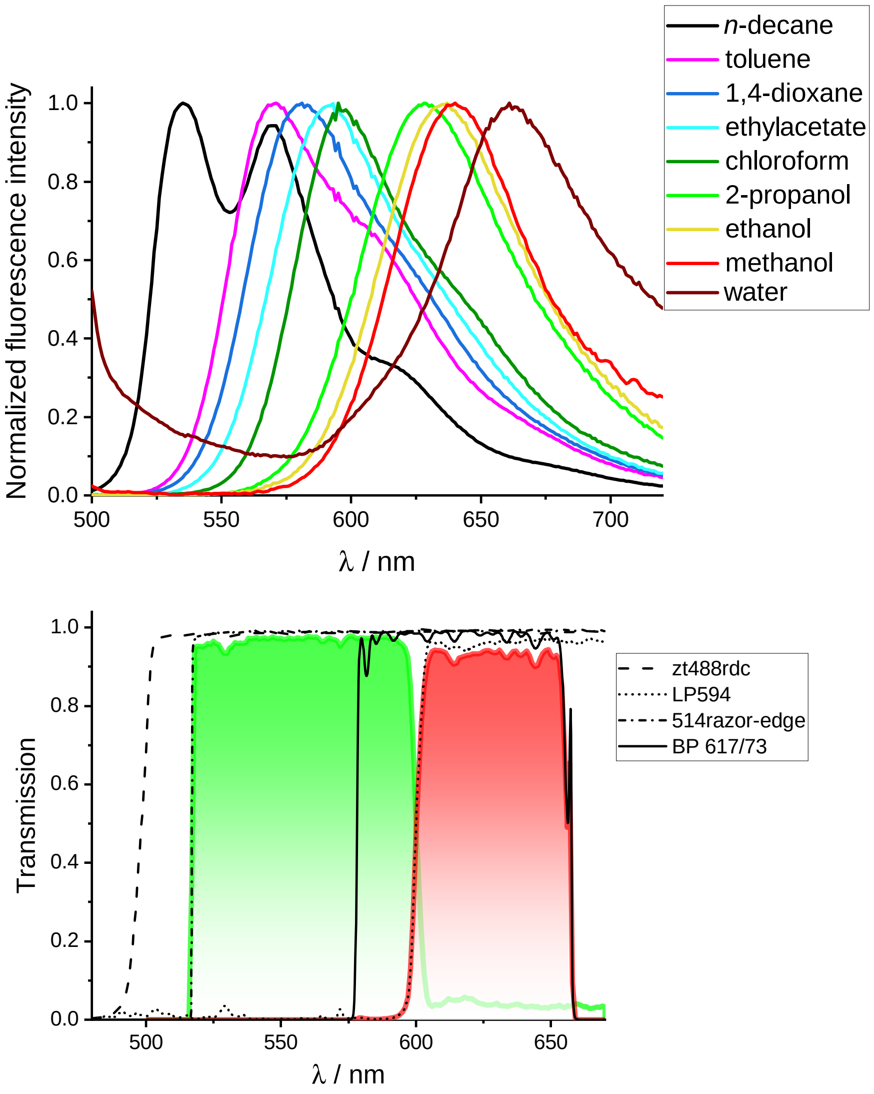


**Figure S6:** (top) Normalized emission spectra of Nile Red in different solvents and (bottom) transmission spectra of different filters used in the widefield-setup including the Optosplit. Combination of the filters results in the long and the short wavelength range shown as the red and green integrales, respectively, over the product of the corresponding filter transmissions.

# 8. Temperature dependent fluorescence spectra of Nile Red in different solvents

The temperature dependent fluorescence spectra of Nile Red in different solvents were obtained on a Jasco FP6500 fluorescence spectrometer with an excitation wavelength of 488 nm. The spectra were corrected with the appropriate wavelength-dependent apparatus function of the spectrometer using the *Calibration Kit* Spectral Fluorescence Standards of the Federal Institute for Materials Research and Testing (BAM, Berlin, Germany) and the software LINKCORR(as in section 7). All spectra (except Nile Red in water) were recorded with low gain, 0.1 seconds response time, scan speed of 200 nm/min and number of accumulations as 1. In case of Nile Red dissolved in water, due to weak fluorescence the gain was set as high and the number of accumulations were set to 10. The other settings were not changed. The Raman bands of water were observed because of a weak fluorescence. These were subtracted by taking blank measurements of only water with the same measurement conditions as in case of Nile Red dissolved in water at different temperatures. In order to compare the wavelength shift in the emission maxima, all spectra were normalized with respect to the intensity

at their respective emission maxima. Figures S7 to S10 represent the normalized fluorescence of Nile Red in different solvents measured at 21, 33, 35, 38, 43 and 53 °C. The temperature range was selected according to the PAINT measurements of microgels.

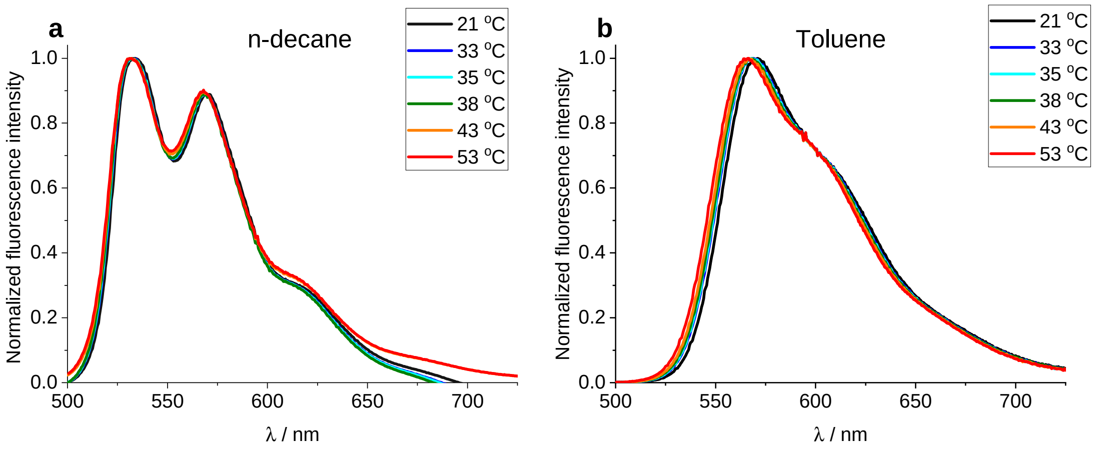


**Figure S7:** Normalized fluorescence spectra of Nile Red in (a) n-decane and (b) toluene at different temperatures as depicted in the legends.

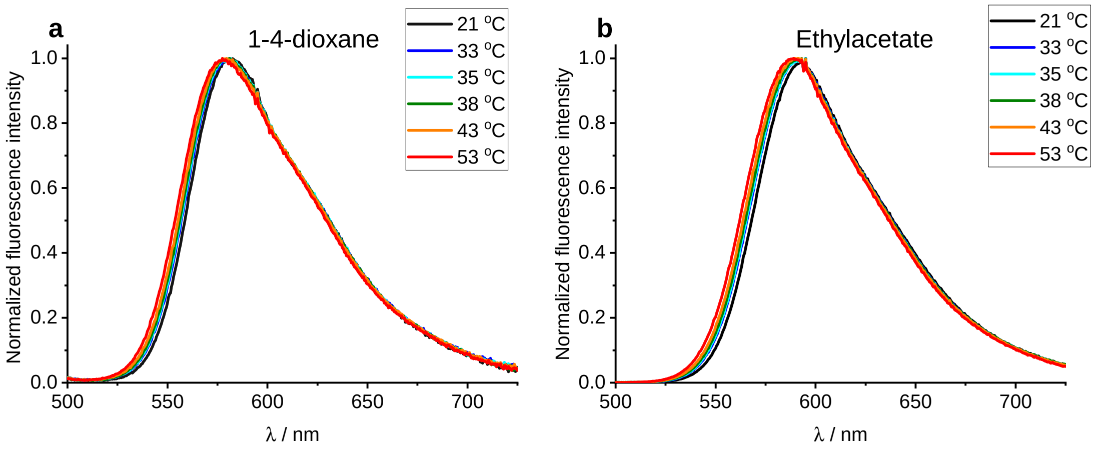


**Figure S8:** Normalized fluorescence spectra of Nile Red in (a) 1-4-dioxane and (b) ethylacetate at different temperatures as depicted in the legends.

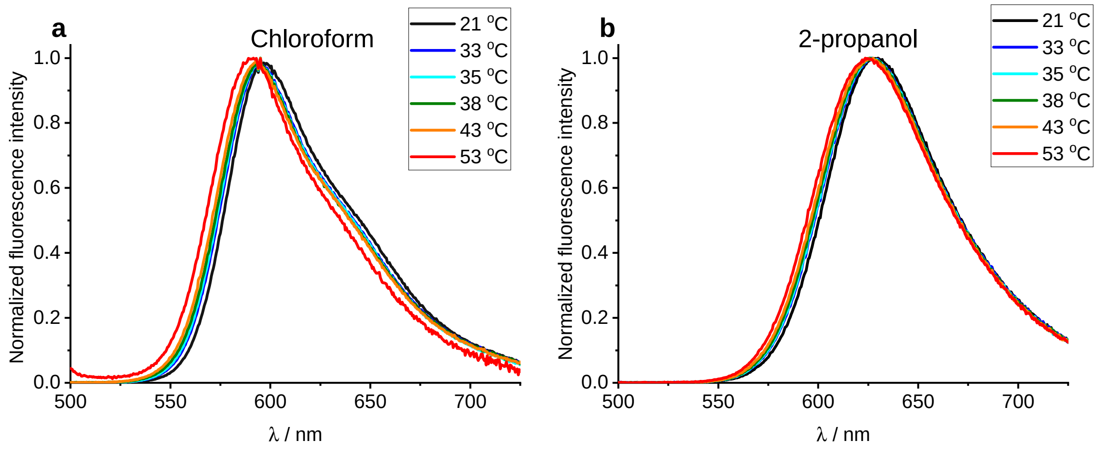


**Figure S9:** Normalized fluorescence spectra of Nile Red in (a) chloroform and (b) 2-propanol at different temperatures as depicted in the legends.

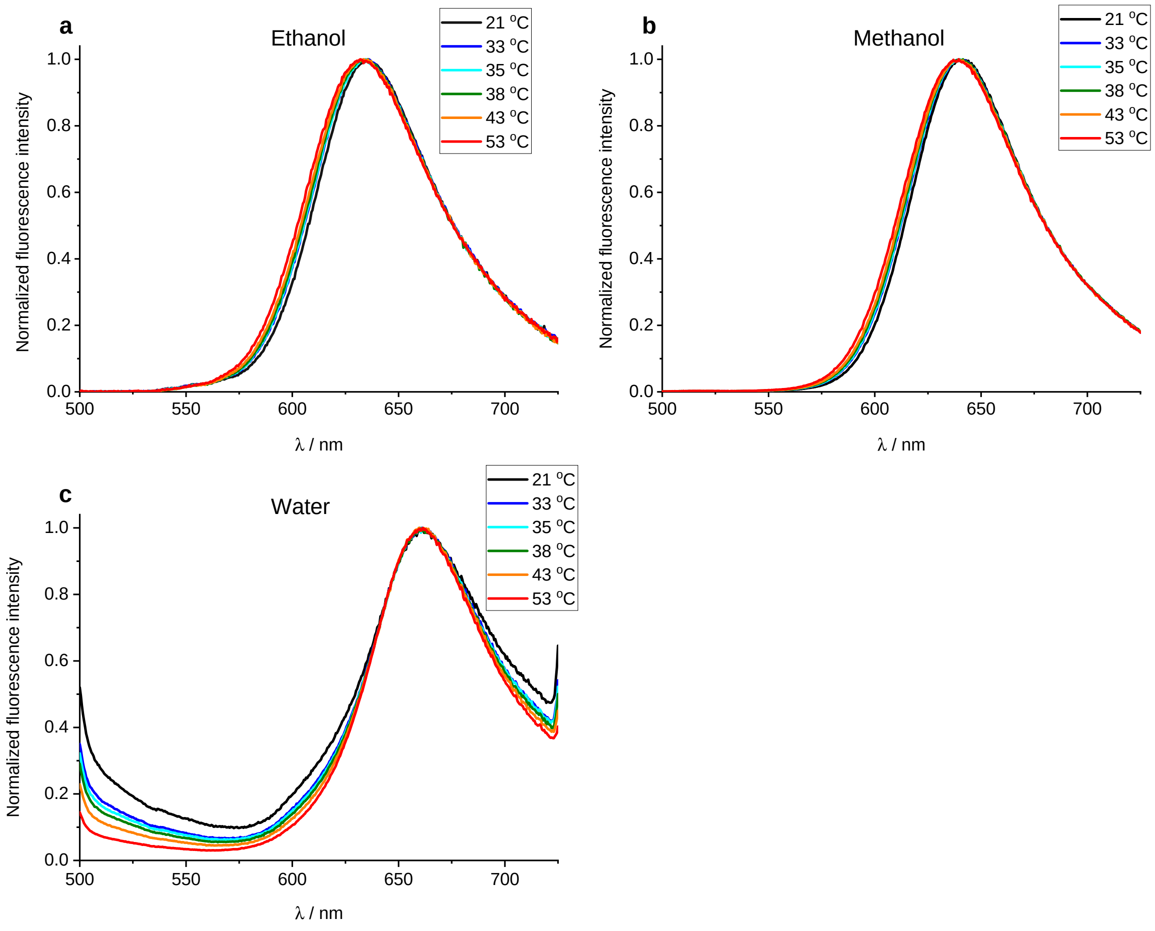


**Figure S10:** Normalized fluorescence spectra of Nile Red in (a) ethanol, (b) methanol and (c) water at different temperatures as depicted in the legends.

The temperature dependent fluorescence properties of Nile Red in various solvents at different temperatures were studied. The hypsochromic wavelength shifts obtained are presented in Table S2. An average hypsochromic shift of approximately 3 nm is obtained in case of these 9 solvents. This shift is not so significant when compared to the wavelength shift as observed for the PAINT measurements of microgels.

**Table S2:** Hypsochromic shift of emission wavelength maxima as observed during temperature dependent fluorescence measurements of Nile Red in different solvents

| Solvents | $\lambda_{max}$ / nm at 21 °C | $\lambda_{max}$ / nm at 53 °C | $\lambda_{shift}$ / nm |
|---|---|---|---|
| n-decane | 533.5 | 532 | 1.5 |
| toluene | 571 | 566.5 | 4.5 |
| 1-4-dioxane | 581 | 577.5 | 3.5 |
| ethylacetate | 595 | 589 | 6 |
| chloroform | 597 | 591.5 | 5.5 |
| 2-propanol | 627 | 623.5 | 3.5 |
| Ethanol | 634.5 | 632 | 2.5 |
| methanol | 640 | 639.5 | 0.5 |
| water | 661 | 661.5 | -0.5 |

# 9. Non-normalized fluorescence spectra of Nile Red in different solvents at different temperatures

In order to compare the fluorescence intensities of Nile Red at different temperatures in each of the solvents used, non-normalized fluorescence spectra were considered and are plotted in the Figures S11 to S14. In case of n-decane, chloroform, ethanol and methanol, a trend of decrease in fluorescence intensities is observed with the increasing temperature. Whereas for water, the trend observed is opposite. In case of dioxane there is an increase in fluorescence intensity which later stays constant at 33 and 35 °C. Beyond 35 °C, there is sudden increase in fluorescence intensity which then stays rather constant until 53 °C. Fluorescence intensity of Nile Red in toluene, ethanol and 2-propanol remains approximately constant with increasing temperature. To circumvent the solvent evaporation, all the spectra measurements were carried out by appropriately sealing the cuvettes with parafilm.

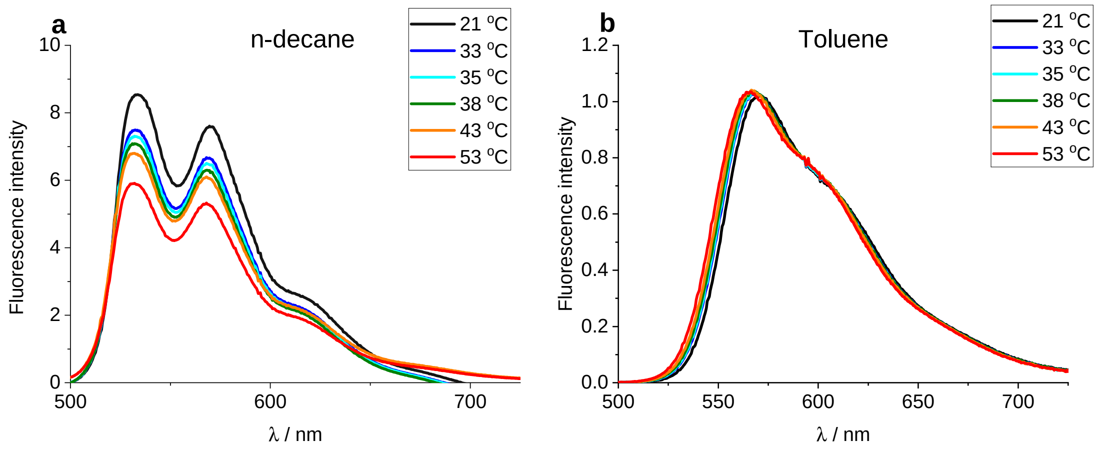


**Figure S11:** Fluorescence spectra of Nile Red in (a) n-decane and (b) toluene at different temperatures as depicted in the legends.

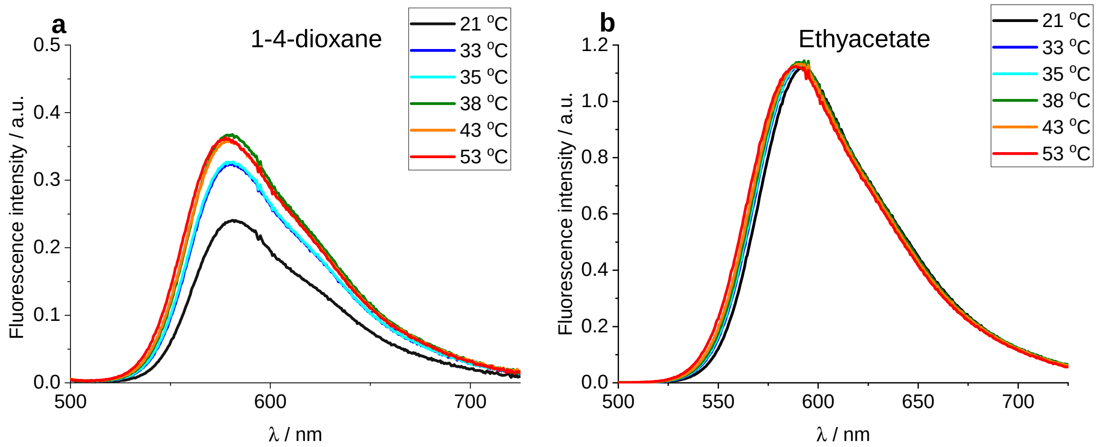


**Figure S12:** Fluorescence spectra of Nile Red in (a) 1-4-dioxane and (b) ethylacetate at different temperatures as depicted in the legends.

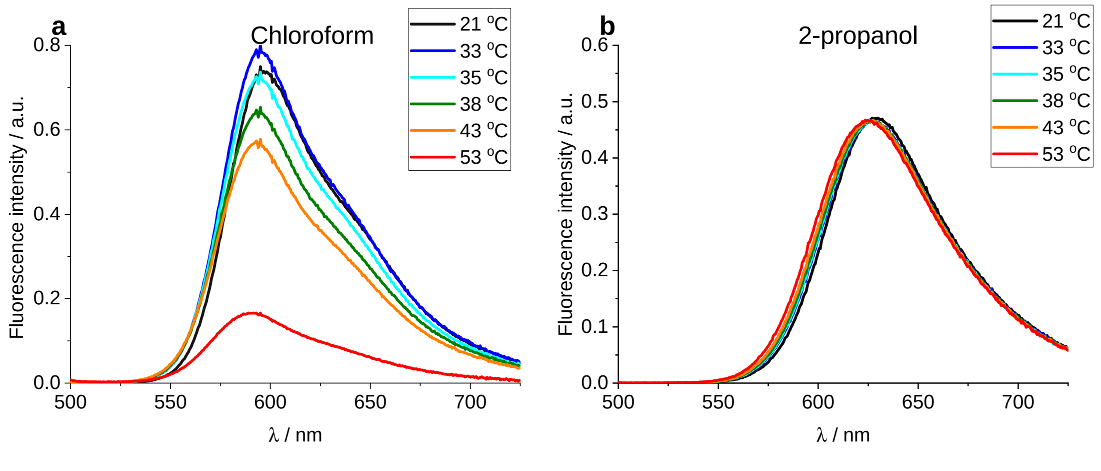


**Figure S13:** Fluorescence spectra of Nile Red in (a) chloroform and (b) 2-propanol at different temperatures as depicted in the legends.

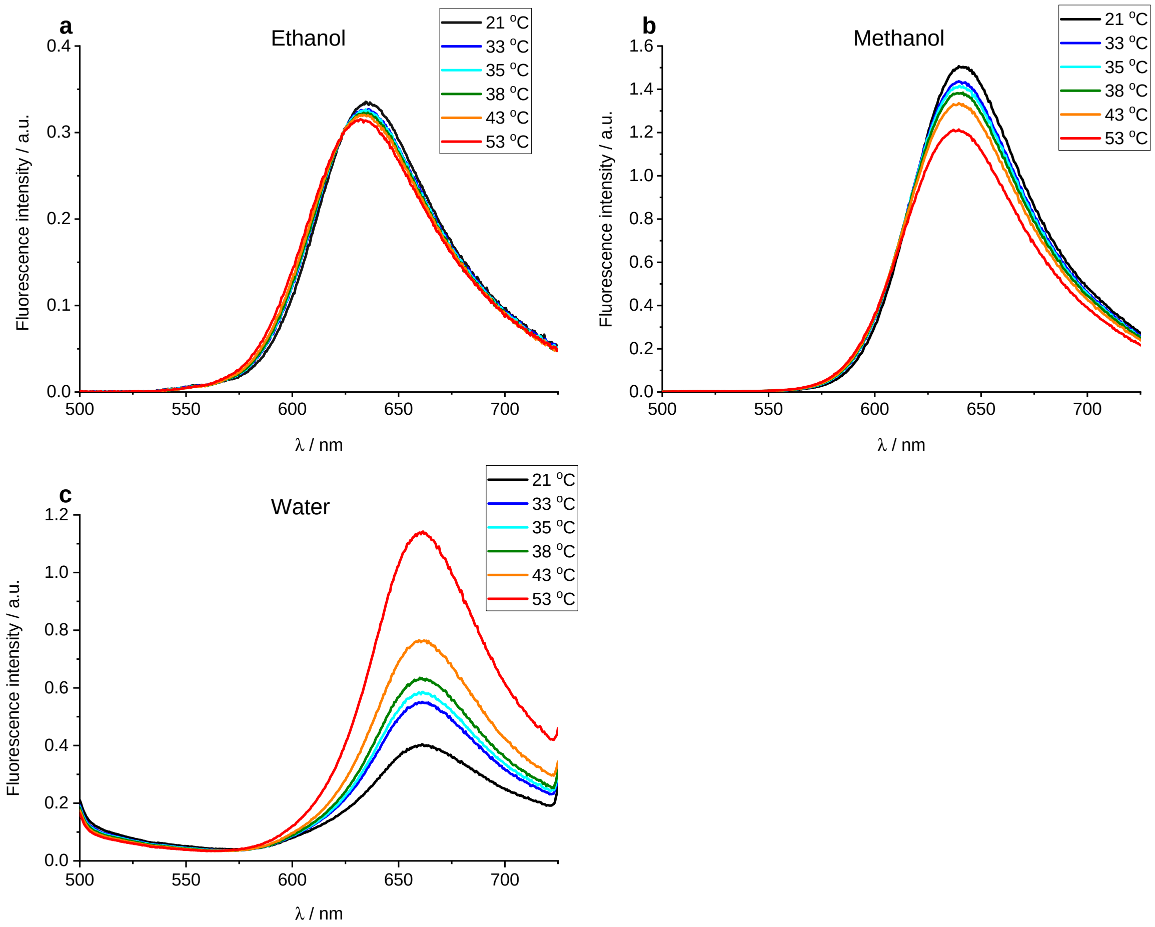


**Figure S14:** Fluorescence spectra of Nile Red in (a) ethanol, (b) methanol and (c) water at different temperatures as depicted in the legends.

## 10. SLS measurements

Static light scattering experiments were performed on a closed goniometer supplied by SLS-Systemtechnik GmbH. In order to minimize multiple scattering and contributions of the structure factor, highly diluted aqueous samples were prepared. To remove small aggregates, all samples were filtered using regenerated cellulose Chromafil syringe filters with a pore size of 0.45 $\mu$m. A wavelength of $\lambda = 407$ nm was used to cover a $q$-range of 0.007 nm$^{-1}$ to 0.04 nm$^{-1}$. The goniometer was connected to a thermostat (Julabo CF40) to control the temperature. Temperature was set to 21 °C, 38 °C or 53 °C. The angle was varied between 15° and 150° in 1° increments. Solvent intensity was measured and subtracted as background. The form factors were imported to FitIt! (O. L. J. Virtanen, FitIt!, 2015. https://www.github.com/ovirtanen/fitit) and fitted using a fuzzy core-shell model. Back-reflection of the cuvette was corrected for all measurements.

The scattering amplitude for core-shell microgels can be described by equation S1.[3][4]

$$A_q = \Delta\rho_{\text{shell}} \cdot V_{\text{shell}} \cdot \varphi_{\text{shell}}(q, R_{\text{out}}, \sigma_{\text{out}}) + (\Delta\rho_{\text{core}} - \Delta\rho_{\text{shell}}) \cdot V_{\text{core}} \cdot \varphi_{\text{core}}(q, R_{\text{in}}, \sigma_{\text{in}}) \tag{1}$$

$\varphi(q, R, \sigma)$ describes the normalized Fourier transformation of the radial density profile:

$$\begin{aligned}\varphi(q,R,\sigma) = \frac{1}{V_n} \cdot \Bigg( \left(\frac{R}{\sigma^2} + \frac{1}{\sigma}\right) \cdot \frac{\cos\left(q \cdot (R+\sigma)\right)}{q^4} + \left(\frac{R}{\sigma^2} - \frac{1}{\sigma}\right) \cdot \frac{\cos\left(q \cdot (R-\sigma)\right)}{q^4} \\ - \frac{3 \cdot \sin\left(q \cdot (R-\sigma)\right)}{q^5 \cdot \sigma^2} - \frac{2 \cdot R \cdot \cos(q \cdot R)}{q^4 \cdot \sigma^2} + - \frac{6 \cdot \sin(q \cdot R)}{q^5 \cdot \sigma^2} \Bigg)\end{aligned} \tag{2}$$

where $V_n = \left(\frac{R^3}{3} + \frac{R\sigma^2}{6}\right)$. The width of core and shell can be calculated from $R_{\text{in}}$ and $R_{\text{out}}$ via $R_{\text{in}} = W_{\text{core}} + \sigma_{\text{in}}$ and $R_{\text{out}} = W_{\text{core}} + 2\sigma_{\text{in}} + W_{\text{shell}} + \sigma_{\text{out}}$. The form factor is proportional to the squared scattering length amplitude as described by equation S3.

$$P(q) \propto A^2(q) \tag{3}$$

The characteristic parameters $W_{\text{core}}$, $\sigma_{\text{in}}$, $W_{\text{shell}}$ and $\sigma_{\text{out}}$ can be calculated from the fitting results and are listed in Table S3.

**Table S3:** Fitting parameters for the core-shell microgel at 21 °C, 38 °C and 53 °C obtained via fits of the form factors using the fuzzy core shell model.

| Temperature | $W_{\text{core}}$ / nm | $\sigma_{\text{in}}$ / nm | $W_{\text{shell}}$ / nm | $\sigma_{\text{out}}$ / nm |
|---|---|---|---|---|
| 21 °C | $111 \pm 4$ | $27 \pm 2$ | $39 \pm 3$ | $9 \pm 3$ |
| 38 °C | $122 \pm 1$ | $0.1 \pm 0.1$ | $6.3 \pm 0.5$ | $19.3 \pm 0.4$ |
| 53 °C | $117 \pm 1$ | $0.7 \pm 0.2$ | $9.4 \pm 0.5$ | $1.2 \pm 0.4$ |

The mean radii of the microgel obtained via SLS are $R_{\text{SLS}} = 214 \pm 12$ nm at 21 °C, $R_{\text{SLS}} = 148 \pm 2$ nm at 38 °C and $R_{\text{SLS}} = 129 \pm 2$ nm at 53 °C. The radius of the core is $138 \pm 5$ nm when both, core and shell, are in their swollen state. When the core collapses, the shell hinders the core to collapse completely, so its size decreases only to $122.5 \pm 0.5$ nm. It is even more decreased when also the shell collapses, reaching a value of $118 \pm 1$ nm.

# 11. PAINT measurements on core-only microgels

For direct comparison with the results of core-shell microgels, we also measured the corresponding core-only microgel consisting only of the PNIPAM core before adding the shell on it. Details of the synthesis can be found in section 5 of the Supporting Information. The results of measurements on these core-only microgels are summarized in the following section in Figure S15 until Figure S18.

The average localization densities of 20 microgels (core-only PNIPAM) at 3 different temperatures are as shown in Figure S15. The axially symmetrical data are presented the same manner as described in the Figure 1 of the main paper.

The 3D distribution of Nile Red labels of a single core-only PNIPAM microgel along with $xy$, $yz$ and $xz$-projections at 21 °C, 33 °C and 53 °C), respectively, is depicted in Figure S16. The sizes of the microgels obtained via PAINT are in agreement with that of the DLS measurements at the respective temperatures. Slight elongation in $z$ direction is due to the localization accuracy of approx. 60 nm as also observed in case of core-shell microgels. The colour code indicates the solvatochromic value as explained in the description of Figure 2 in the main paper.

Figure S17 depicts the averaged super-resolved solvatochromic image at 3 different temperatures 21 °C, 33 °C, 53 °C obtained by employing a similar approach as in the case of Figure 3 in the main paper.

Figure S18 represents the radial solvatochromic values and the radial distribution plots of core-only PNIPAM microgels based on the same approach as outlined in Figure 4 of the main paper.

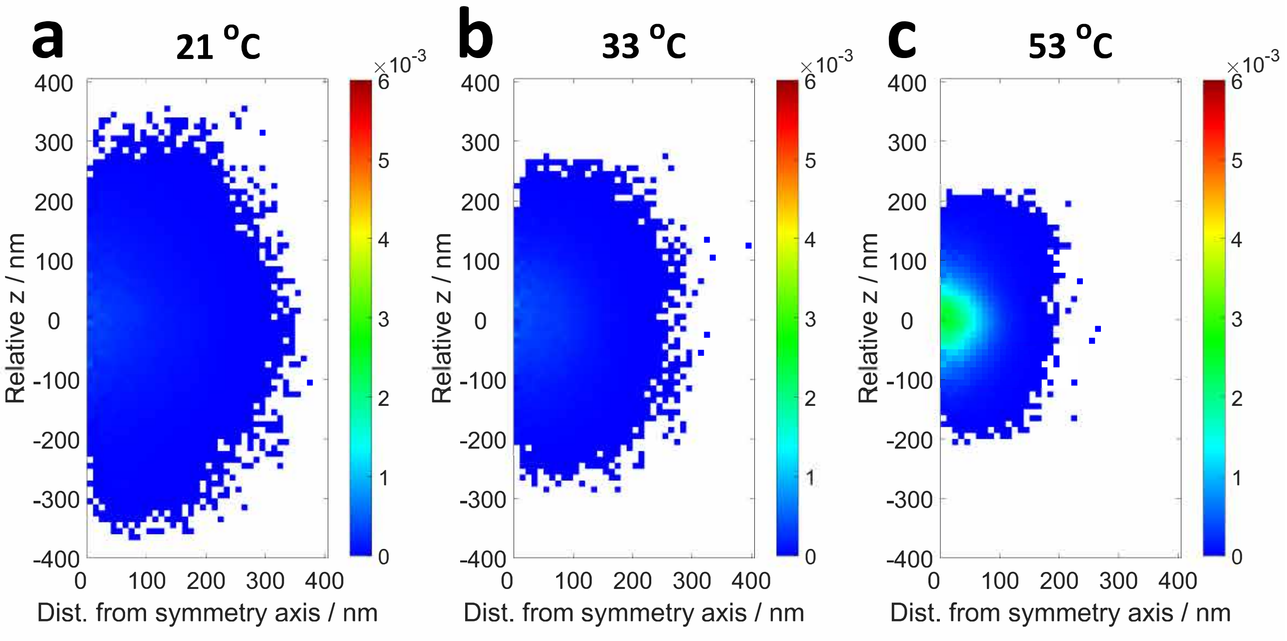


**Figure S15:** Average localization densities of 20 core-only PNIPAM microgels at a temperature of a) 21 °C, b) 33 °C, c) 53 °C.

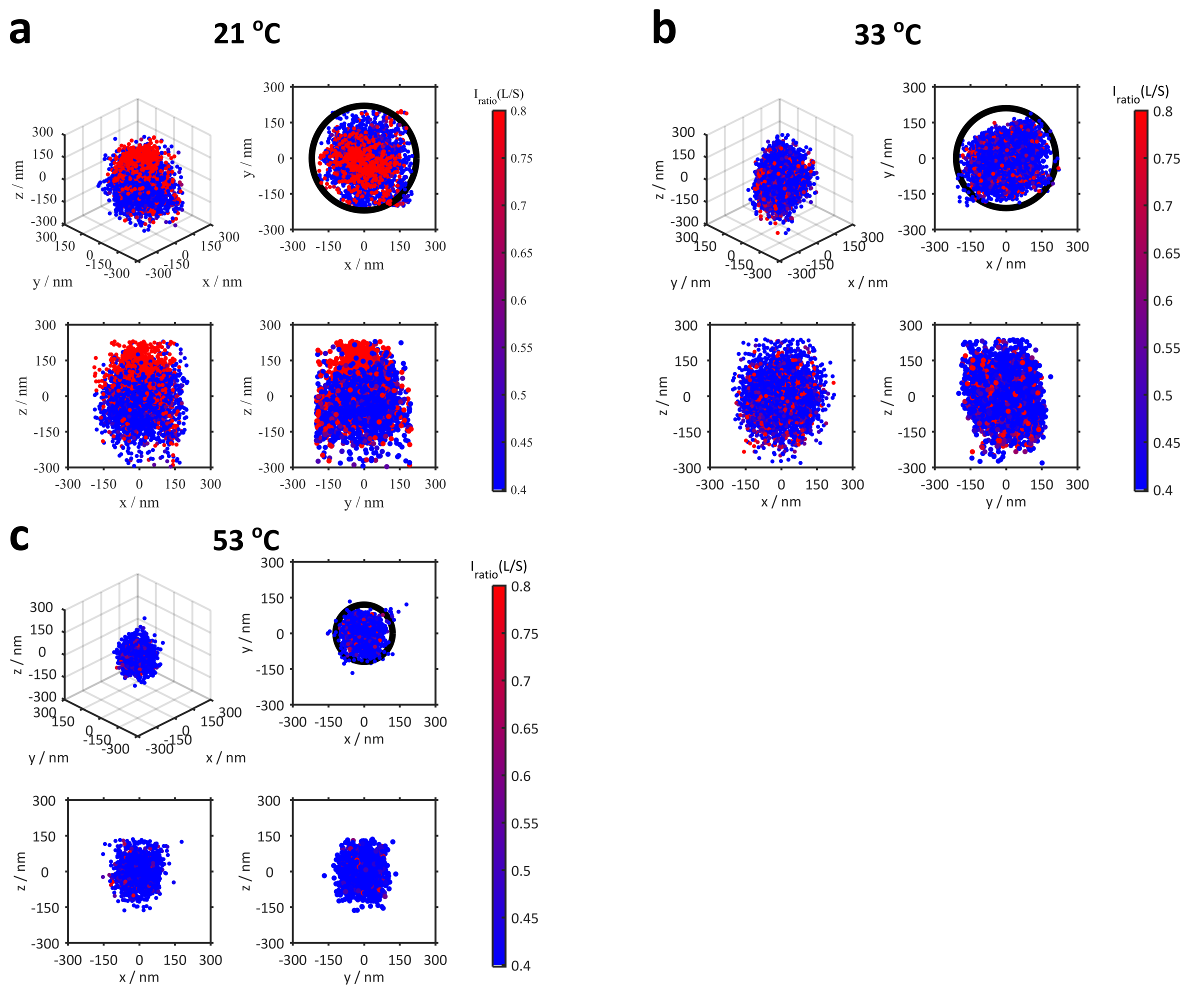


**Figure S16:** 3D distribution of the localizations of a single representative core-only PNIPAM microgel (a) at 21 °C and (b) at 33 °C, (c) at 53 °C, respectively. A full 3D view as well as projections to the $xy$, $xz$ and $yz$ planes are shown. The colours represent the solvatochromic values expressed by the ratio between the long wavelength (> 594 nm) and the short wavelength (< 594 nm) channel with the scaling from 0.4 to 0.8. (see colour bar) The circle in the $xy$-projection represents the hydrodynamic radius of the microgel determined via DLS which is 220 nm for (a), 210 nm for (b) and 120 nm for (c).

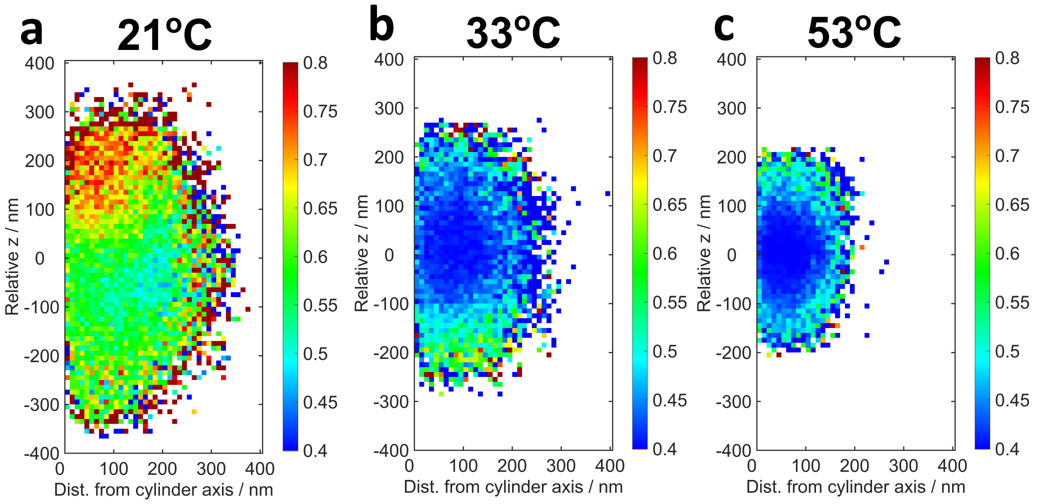


**Figure S17:** 3D distribution of the solvatochromic values of localizations in core-only PNIPAM microgels. The colours represent the solvatochromic values expressed by the ratio between the long wavelength (> 594 nm) and the short wavelength (< 594 nm) channel with the scaling from 0.4 to 0.8. Solvatochromic values averaged over 20 microgels at a) 21 °C, b) 33 °C, c) 53 °C of core only PNIPAM microgels.

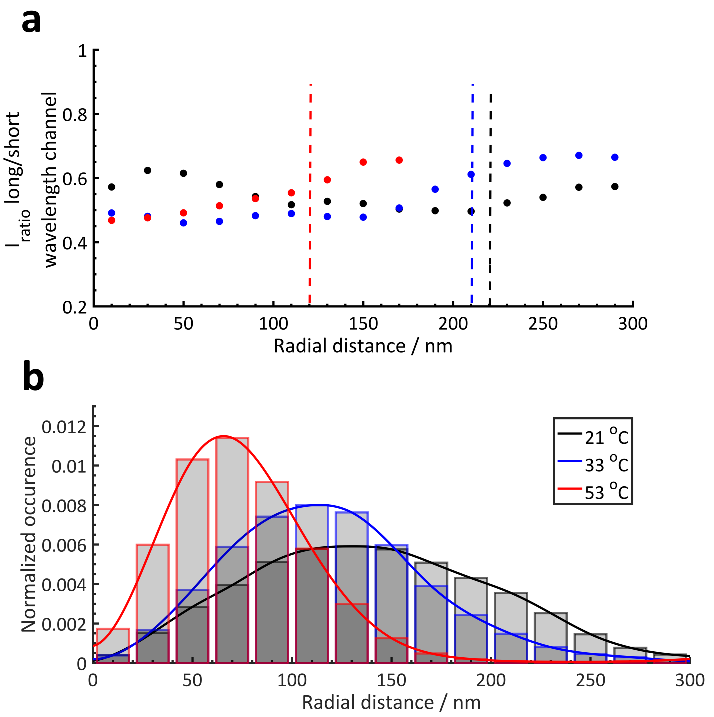


**Figure S18:** (a) Radial solvatochromic values of core-only (PNIPAM) microgels at 3 different temperatures viz. 21 °C, 33 °C, 53 °C. Dashed lines indicate the corresponding hydrodynamic radii determined via DLS measurements. (b) Normalized radial distributions of the microgels at different temperatures. The coloured lines are guides to the eyes.

## 12. Comparison of the core-shell-microgels with the corresponding core-only microgels

Radial solvatochromic values of core shell microgels are compared with that of corresponding core-only microgels in Figure S19. The radial solvatochromic values for the core-shell microgels are depicted for 6 different temperatures as the PNIPAM core and PNIPMAM shell have VPTTs of 32 °C and 42 °C, respectively, thus covering also the intermediate range of temperatures. In case of core-only microgels made up of PNIPAM, only 3 temperatures are considered as these microgels already collapse at 32 °C.

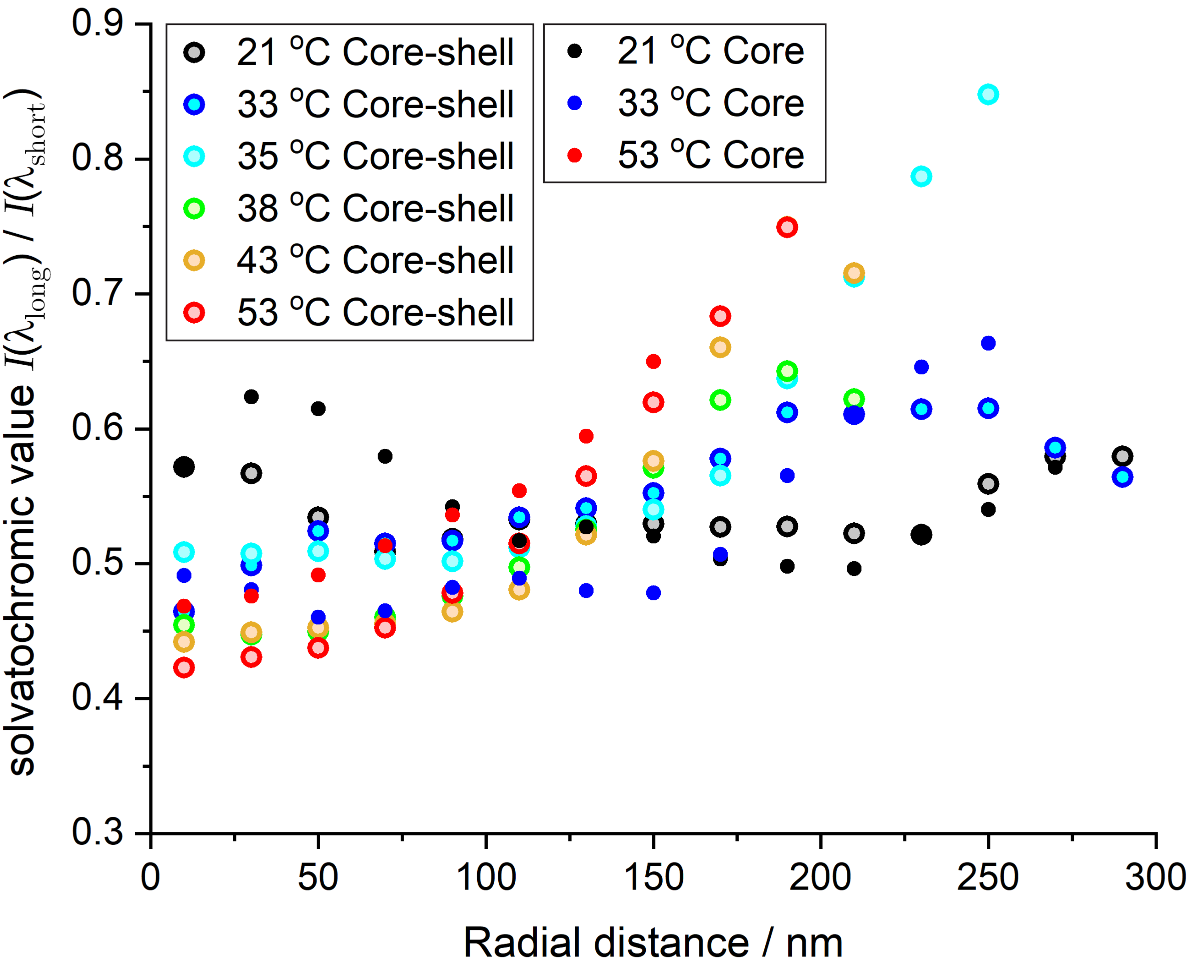


**Figure S19:** Radial solvatochromic values of core-shell (PNIPAM core and PNIPMAM shell) and core-only (PNIPAM) microgels at different temperatures.

### 12.1. Boxplot

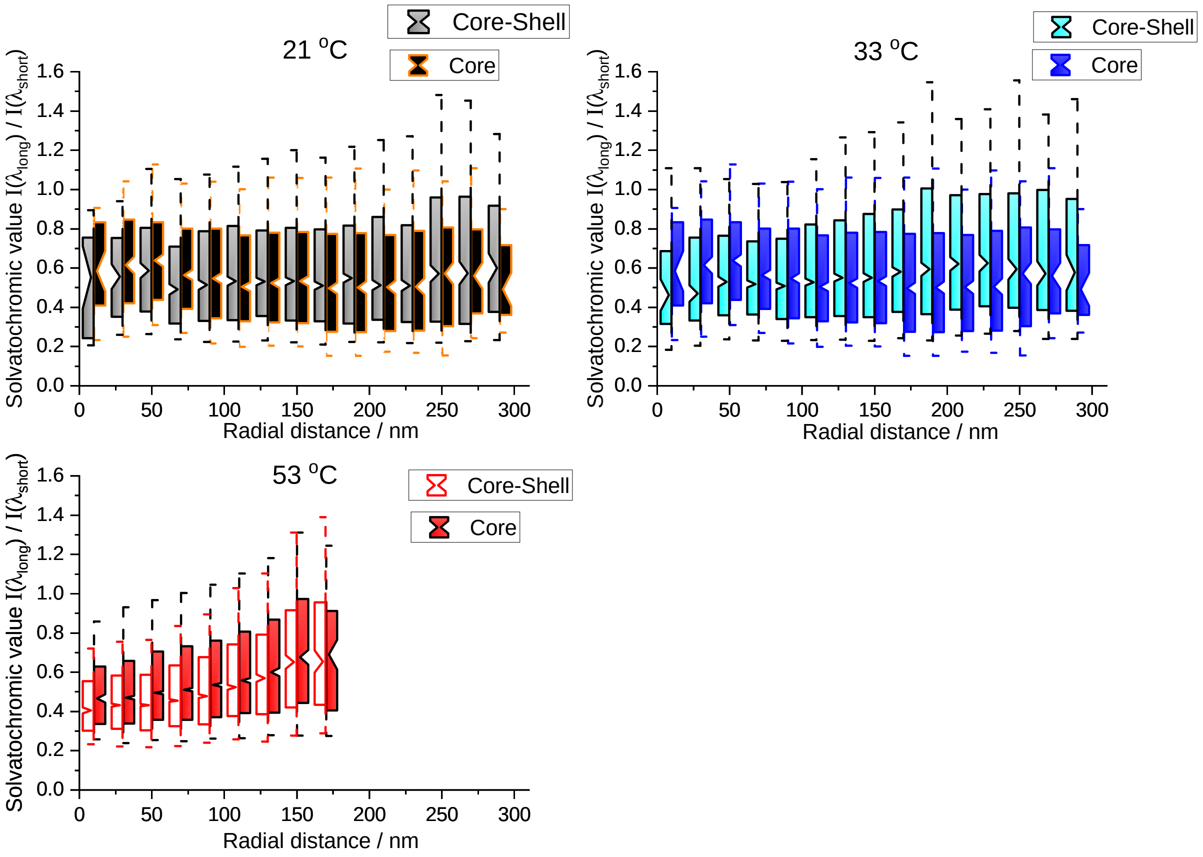


**Figure S20:** Radial solvatochromic values presented as boxplots of core-shell (PNIPAM core and PNIPMAM shell) and core-only (PNIPAM) microgels at 21, 33 and 53 °C. The whisker range is from 10 to 90 percentile. The notch represents 95% confidence interval of the respective median value.

## 13. Supporting movies

The following movies are presented in the Supporting Material:

1. Calibration movies
   - Movie 1 shows a scan over the entire field of view of TetraSpeck™ Microspheres (0.1 µm, blue/green/orange/dark red fluorescence, ThermoFisher scientific) in $x$ and $y$ directions in a snake like pattern. The Optosplit 2 divides the image in a longer wavelength channel (upper part of the images) and a shorter wavelength channel (lower part of the images). The movie was captured with an exposure time of 0.05 s and electron multiplying mode with a gain of 100 and readout rate of 17 MHz at 16 bit. 1071 frames were acquired for the entire scan.
   - Movie 2 shows a $z$ scan over the tetraspeck beads within a $z$ range of 1 µm and with 20 nm step size. The movie was captured with the same camera setting as in case of Movie 1 except for the number of frames which was 50.
2. Raw movies of PAINT measurements at different temperatures. Movie 3, 4, 5, 6, 7, and 8 consist of 100 frames of the raw movies of rhe PAINT measurements at 21 °C, 33 °C, 35 °C, 38 °C, 43 °C, and 53 °C, respectively. For all theses measurements, a laser power density of 5.3 kW/cm2, an exposure time of 5 ms and electron multiplying mode setting with a gain of 200 were used. The number of frames recorded per area of the sample varied between 60,000 to 120,000.
3. Presentation of single Nile Red localizations with colour-coded polarity of individual microgels at different temperatures. Movies 9, 10, 11, 12, 13, 14 are the distributions of localizations of individual microgels at 21 °C, 33 °C, 35 °C, 38 °C, 43 °C, and 53 °C, respectively. The colour code changes from blue representing the lower polarity with solvatochromic value below 0.5 to red representing the higher polarity with solvatochromic value above 1.0.

[1] B. Huang, W. Wang, M. Bates and X. Zhuang, *Science*, 2008, **319**, 810–813.
[2] A. P. Gelissen, A. Oppermann, T. Caumanns, P. Hebbeker, S. K. Turnhoff, R. Tiwari, S. Eisold, U. Simon, Y. Lu, J. Mayer, W. Richtering, A. Walther and D. Wöll, *Nano Lett.*, 2016, **16**, 7295–7301.
[3] I. Berndt, J. S. Pedersen and W. Richtering, *Angew. Chem. Int. Ed.*, 2006, **45**, 1737–1741.
[4] M. Brugnoni, A. Scotti, A. A. Rudov, A. P. H. Gelissen, T. Caumanns, A. Radulescu, T. Eckert, A. Pich, I. I. Potemkin and W. Richtering, *Macromolecules*, 2018, **51**, 2662–2671.
